# Functional Output Regression for Machine Learning in Materials Science

**DOI:** 10.1021/acs.jcim.2c00626

**Published:** 2022-10-10

**Authors:** Megumi Iwayama, Stephen Wu, Chang Liu, Ryo Yoshida

**Affiliations:** † Department of Statistical Science, The Graduate University for Advanced Studies, Tachikawa190-8562, Japan; ‡ Production Management Headquarters, Process Technology Division, Daicel Corporation, Himeji671-1283, Japan; § Research Organization of Information and Systems, The Institute of Statistical Mathematics, Tachikawa190-8562, Japan; ∥ Research and Service Division of Materials Data and Integrated System, National Institute for Materials Science, Tsukuba305-0047, Japan

## Abstract

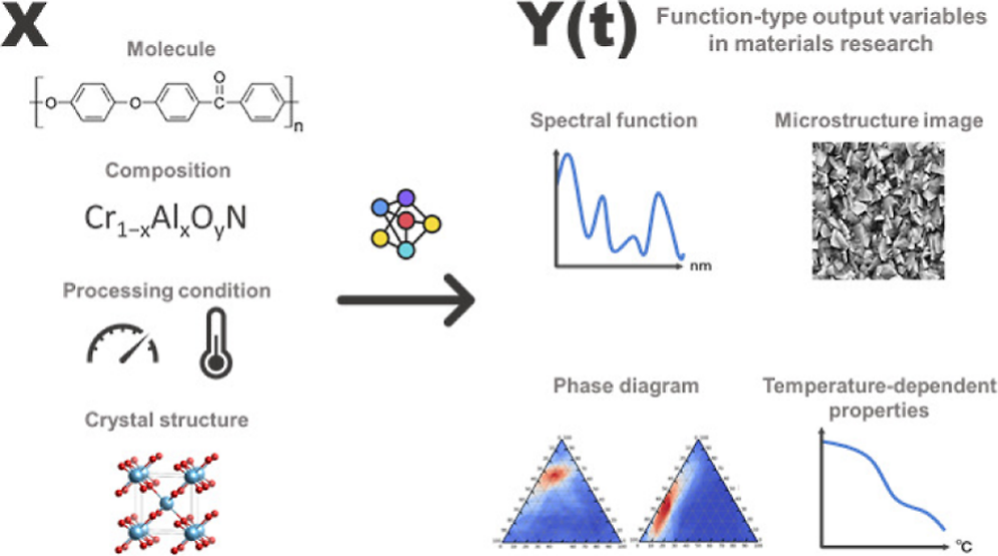

In recent years, there has been a rapid growth in the use of machine learning in material science. Conventionally, a trained predictive model describes a scalar output variable, such as thermodynamic, electronic, or mechanical properties, as a function of input descriptors that vectorize the compositional or structural features of any given material, such as molecules, chemical compositions, or crystalline systems. In machine learning of material data, on the other hand, the output variable is often given as a function. For example, when predicting the optical absorption spectrum of a molecule, the output variable is a spectral function defined in the wavelength domain. Alternatively, in predicting the microstructure of a polymer nanocomposite, the output variable is given as an image from an electron microscope, which can be represented as a two- or three-dimensional function in the image coordinate system. In this study, we consider two unified frameworks to handle such multidimensional or functional output regressions, which are applicable to a wide range of predictive analyses in material science. The first approach employs generative adversarial networks, which are known to exhibit outstanding performance in various computer vision tasks such as image generation, style transfer, and video generation. We also present another type of statistical modeling inspired by a statistical methodology referred to as functional data analysis. This is an extension of kernel regression to deal with functional outputs, and its simple mathematical structure makes it effective in modeling even with small amounts of data. We demonstrate the proposed methods through several case studies in materials science.

## Introduction

Recently, there has been a growing trend to use machine-learning techniques to accelerate the process of designing and creating new materials in various domains of material science. Conventionally, machine-learning models are used to rapidly perform high-throughput virtual screening across millions or billions of candidate materials that span an enormous search space.^1−5^ In general, a model describes physicochemical, electronic, thermodynamic, or mechanical properties as a function of the input materials, which are given in various forms, such as small- or macro-molecules, crystalline systems, chemical or raw material compositions, and their mixtures. To put the task into a machine-learning framework, such a non-numeric variable needs to be transformed into a fixed-length numeric vector called a *descriptor*, which represents the compositional or structural features of the given material.^5−16^ Under the supervision of given data, a model is trained to learn the mapping from the vectorized features to their respective properties. In this workflow, the feature representation of the input materials plays a key role in boosting the predictive power.

There is a great deal of prior work on transforming material features into numeric vectors and constructing regression models or classifiers that represent the mapping from vectorized input materials to their output properties. A class of descriptors, referred to as molecular fingerprints, has long been studied in chemical informatics, which converts a chemical structure or molecular graph into an integer-valued vector according to the presence or absence or the number of occurrences of a particular chemical fragment, in which hundreds or thousands of fragments are considered.^10−14^ Another type of molecular descriptor employs a quantitative representation of the topological or physicochemical features of a molecular system.^17−20^ Chemical composition can be considered as a set variable consisting of a variable number of element species and their contents. There are a large volume of previous studies on the representation of such compositional features.^5−9^ A crystal structure is typically vectorized by encoding the local structural environments of each atom and the neighboring relations of constituent atoms in a unit cell.^7−9,21^ In recent years, there has also been an increasing trend in treating a material structure as a graph and in modeling its properties using graph neural networks (NNs).^22−24^ A natural representation of the chemical structure is created on a labeled graph. A periodic configuration of atoms in a crystalline system can also be translated into a graph called a crystal graph, which represents the coordination of constituent atoms in infinitely arranged unit cells.^22^ In addition, when predicting the properties of a composite system from its microstructure, it is natural to treat the microstructure as an image. In the study of composite materials, scanning electron microscopy (SEM) and transmission electron microscopy (TEM) are widely used to observe the surface or interfacial structure of the fabricated materials. By treating the microstructure as an image, supervised learning can be performed by regressing real-valued output properties onto the space of the microstructure images, as in computer vision and image recognition.^25,26^ Other representation methods have also been investigated for various material systems, such as topological feature representation of disordered material systems using persistent homology,^27^ identification of multi-component materials based on the spectral function of powder X-ray diffraction,^28^ and prediction of reaction outcomes in organic synthesis based on string representations of product and reactant molecules.^29,30^

As mentioned above, most previous studies have considered the ordinary problem setting of supervised learning, where an input variable is given as a relatively high-dimensional vector encoding material features, and the output is a scalar or low-dimensional real-valued vector, for example, a few sets of physicochemical properties or a class label indicating structural species or the level of physical features. On the other hand, there are many potential problem settings in material science, where the output variable is inherently ultra-high-dimensional or multidimensional (e.g., a functional-type output). However, the methodology of supervised learning in such scenarios has not been well studied. For example, in the task of predicting the ultraviolet–visible (UV–vis) absorption spectra of molecules, the input variable is given by a vectorized molecular structure, and the output variable is given as a function defined on the domain of wavelengths that represents the optical absorbance.^31^ In the study of composite materials, it is important to qualitatively and quantitatively understand the influence of processing conditions such as temperature, pressure, and composition on the resulting microstructures. SEM and TEM are commonly used to examine microstructures. If we formulate the problem within a framework of supervised learning, the input is a real-valued vector encoding the processing condition and composition, and the output is given by an intensity matrix representing the grayscale microscopic image. This is a regression problem for multidimensional functional output variables. Alternatively, the problem can be reduced to an image generation task in computer vision. To solve such problems, various types of deep generative models, such as the conditional GAN (cGAN)^32^ and encoder–decoder networks,^33^ can be applied. In fact, there have been several previous studies in which cGAN was applied to the prediction of microstructures, as described above,^34−36^ and an encoder–decoder model was applied to predict the UV–vis absorption spectra of organic molecules.^31^ In addition, in statistical science, regression methods for functional output variables have long been studied in the context of functional data analysis,^37^ which may also be applicable to solving the aforementioned problems.

In this study, we consider two unified frameworks for multidimensional functional output regression that can cover various potential applications in material science. The first approach employs cGAN, inspired by the study of microstructure images in Banko et al.^34^ However, because cGAN has training instability in the adversarial learning process and weakness to limited amounts of data, we developed another framework, a statistical modeling that relies on the methodology of functional data analysis for functional output variables. The present model can be viewed as an extension of kernel regression to handle functional output variables and has a simpler mathematical form than the cGAN model architecture. As shown later, the method exhibits outstanding predictive performance even in cases where only a small amount of data is available. We demonstrate these two methods using three case studies. In the first two case studies, the optical absorption spectra of organic molecules in two different regions of UV–vis (170–780 nm) and near-infrared wavelength (NIR: 780–2500 nm) were predicted. The output variable is a spectral function in the wavelength domain. The number of training instances is approximately over 900 for the former case, whereas for the latter, the amount of data is limited as the number of training molecules is approximately 60. The objective of the third example is to predict the electron microscopic image of the microstructure for any given composition and processing conditions in the fabrication of thin-film composite materials. With these applications, we demonstrate the potential predictive ability of the proposed methods on small amounts of training data. We compare ordinary regression, which predicts a scalar output variable with a pre-quantified spectral feature,^38^ with the present methods predicting the whole function directly, and show the superiority of the latter and its statistical mechanisms in relation to multitask learning.^39,40^ The Python codes used in the case studies were distributed.^41^

## Preliminary

The present study deals with a supervised learning problem, in which the input variable  is a *p*-dimensional descriptor vector and the output variable  is given by a real-valued function of *X* and an additional argument . Argument *t* corresponds to a coordinate in image space or to a wavelength at which the spectral function is defined. In the following sections, we describe potential applications in material science.

### Spectral Prediction

Molecules undergo temporal transitions from their ground states to higher-energy excited electronic states in response to the absorption of light, such as UV–vis or NIR. The absorption wavelength is proportional to the inverse of energy. The absorbance spectrum, which represents the intensity of optical absorption as a function of wavelength, is determined by the excitation energy levels and the transition probabilities of the electronic states in a molecular system. Accurately predicting molecule-specific absorbance spectra is highly beneficial for various applications, such as the design of organic light-emitting diodes,^1,42^ organic photovoltaic cells,^43^ and UV filters.^44^ Usually, absorption peak wavelengths are predicted from the excited states of electrons obtained ab initio, for example, by performing time-dependent density functional theory calculations.^45^ However, owing to the high computational cost, first-principles calculations are not useful for exhaustive molecular screening. Furthermore, while the location and intensity of a peak wavelength can be estimated ab initio, other functional features of the full spectrum, such as the full width at half maximum and absorbance integration in a wavelength interval, cannot be determined.

**Figure 1:**
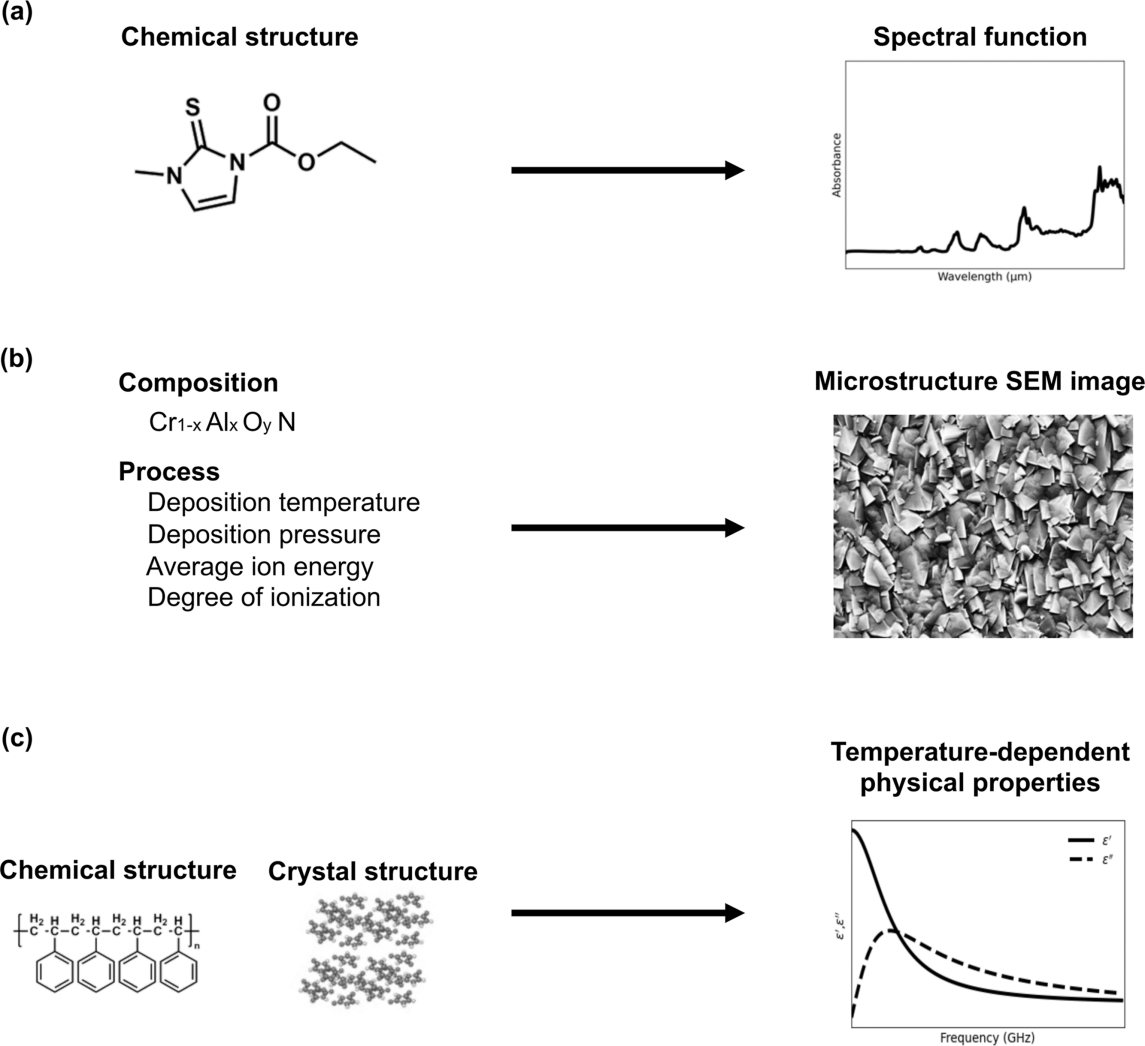
Three potential applications formulated as the problem of functional output regression: (a) prediction of optical absorption spectra based on the chemical structure of an organic molecule, (b) prediction of microstructure images of composite materials based on processing conditions and compositional features, and (c) prediction of frequency-dependent physical properties.

Here, we address the problem of spectral prediction using a fully data-driven approach that does not rely on ab initio calculations (Figure 1a). The input variable of model *f* consists of a descriptor vector *X* and a wavelength , where the descriptor *X* encodes the structural and compositional features of the input molecule. The output variable is the spectral function *Y*(*X*,*t*) of the optical absorbance with respect to varying molecules and wavelengths. In summary, with measurement noise ϵ, the model can be expressed as
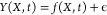
1

Suppose that for each of the *n* observed molecules {*X*_*i*_|*i* = 1, ..., *n*}, the absorption spectrum *Y*(*X*,*t*) is measured over *m* discretized wavelengths {*t*_*j*_|*j* = 1, ..., *m*}. In this study, it is assumed that the observation points of the wavelength are common to all molecules: *t*_*j*_ is independent of the index *i* of the molecule. When the observed wavelengths vary across the molecules, one can obtain a series of complete data with the same observation points by smoothly interpolating the missing data points. The task then comes down to a regression problem for the high-dimensional vector-valued output, which is modeled by
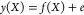
2where *y*(*X*)^T^ = (*Y*(*X*,*t*_1_), ..., *Y*(*X*,*t*_*m*_)), *f*(*X*)^T^ = (*f*(*X*,*t*_1_), ..., *f*(*X*, *t*_*m*_)), and *e*^T^ = (ϵ_1_, ..., ϵ_*m*_). As mentioned above, it is assumed that there are no missing data or that the missing data in the direction *t* have been interpolated beforehand based on a sufficient amount of observed data. However, the functional output kernel regression, which will be shown later, can naturally perform training with no special treatment for missing data, even if the observation points of *t* are quite sparse.

Machine learning for predicting the optical absorption spectra of molecular systems has not been extensively studied. To clarify the contribution of our work, we consider the recently published work of Urbina et al.^31^ that relied on Seq2Seq^33^ and its variant encoder–decoder architectures with a built-in attention mechanism. Seq2Seq, which is widely used in natural language processing, was utilized to learn mapping from tokenized SMILES strings^46^ or pre-defined molecular descriptors of input chemical structures to the absorption spectra. On the other hand, we introduce a much simpler statistical model, the functional output kernel regression, aimed at stabilizing the learning process by reducing over-parameterization and achieving a high prediction accuracy even in cases where sufficient amounts of data are unavailable for model training. Another distinctive feature of the present method is its high degree of interpretability, which directly describes the occurrence of a peak in a specific wavelength range in relation to the presence or absence of molecular fragments encoded in the descriptor *X*.

In this study, we focus on the advantages of directly predicting the entire spectral function, rather than predicting a pre-defined univariate functional feature, such as the wavelength of maximum absorbance λ_max_. In the experiments reported later, it was confirmed that the prediction accuracy of λ_max_ calculated from the predicted spectral function often significantly exceeds that of the conventional univariate output regression directly trained with the pre-quantified λ_max_. It should be noted that high-dimensional output variables *y*(*X*) = (*Y*(*X*,*t*_1_), ..., *Y*(*X*,*t*_*m*_)) are closely related. Learning a single model for multivariate outputs simultaneously can be considered a type of multitask learning. In multitask learning, multiple related tasks are learned simultaneously, allowing the model to recognize common mechanisms among target tasks and consequently improve the prediction accuracy of each task.^39^ A similar learning mechanism is expected to work in regression with high-dimensional output variables.

### Microstructure Image Prediction

Microstructures with varying morphologies, volume fractions, and grain-size distributions can be designed by controlling the composition of the material species and processing conditions.^34−36^ Here, we consider the problem of predicting the microstructures for any given compositional and processing parameters. To treat the microstructure as a model output, we use an image obtained by optical or electron microscopy. In the development of composite materials, SEM or TEM has widely been used to analyze the surface and morphologies. Practically, for example, we aim to improve the mechanical properties of a material by controlling the composition and temperature to obtain a finer and more homogeneous grain structure. By defining a microstructure as an image, we can address the machine-learning task by utilizing various well-established techniques in image recognition and computer vision.^34−36^ Specifically, the input variable *X* is given by a real-valued vector representing the compositional and processing parameters, and the output *Y*(*X*,*t*) is defined as a microscopic image that takes a matrix or tensor form for a grayscale or color image, respectively. The variable *t* represents a two- or three-dimensional image coordinate, and its support is discretized into pixel or voxel positions. The model can therefore be written in the form of a multidimensional vector regression, in the same way as the model for the spectral function described above (Figure 1b). Alternatively, in the context of computer vision research, this task can be regarded as machine learning for conditional image generation.

### Other Potential Applications

There are many other applications in material science where the prediction of functional outputs is applicable. Many physical properties are determined by temperature, pressure, and frequency in an external electric field (Figure 1c). The dielectric properties of a material, that is, the dielectric constant or dielectric loss tangent, are given as a function of frequency and temperature.^47^ In this case, *t* comprises the two variables. In addition, various polymeric properties, such as the specific volume, linear expansion coefficient, elastic modulus, specific heat, and thermal conductivity, are also dependent on temperature. From the observed transition points in the temperature dependence curve, the glass transition temperature, crystal melting point, and crystallization temperature are calculated. For the imaging analysis of materials, a wide variety of microscopes such as SEM, TEM, and optical microscopes are commonly used, depending on the size of the object to be observed. Various technologies of three-dimensional measurements have been established to analyze the internal structures of materials. For example, three-dimensional TEM allows us to observe the morphology of objects ranging in size from tens to hundreds of nanometers in three dimensions.^48^ X-ray computed tomography (CT) is a non-destructive technique that is often used in medical applications. With this method, we can observe microstructures ranging from a few micrometers to millimeters in size without polishing or etching the sample surface.^49^ In addition, X-ray CT is a non-destructive inspection method that can be used to measure the fracture process of a material and the change in the material structure in response to heating in four dimensions (three-dimensional space plus time). Furthermore, synchrotron X-ray CT using high-brilliance synchrotron radiation can non-invasively measure the inner structure of materials with a high resolution of several hundred nanometers to several micrometers, even in metals where X-ray penetration is difficult.^50^ In principle, the proposed regression method can be applied to a wide range of high-dimensional functional data.

## Methods

In this paper, we present two different regression methodologies for multidimensional functional outputs: deep generative modeling using adversarial learning and deep kernel regression for functional outputs. The former, originally developed for image-generation tasks,^34^ is introduced to solve the two research subjects. The latter is a newly developed method for overcoming the limited learning performance of the deep generative models.

### Conditional GAN

**Figure 2:**
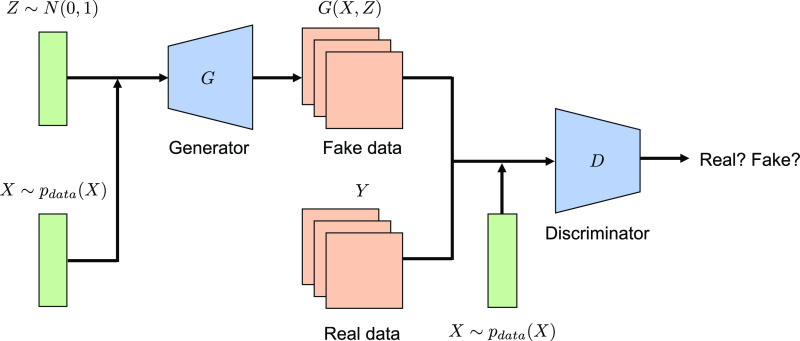
Model architecture of cGAN. The generator (*G*) represents a mapping from composition/process conditions or molecular descriptor *X* to a microstructure image or optical absorption spectral function. The additional input *Z* denotes a Gaussian random noise. The discriminator (*D*) determines, given an image or spectral function in addition to the conditional variable *X*, whether it is real or fake. Model architectures are detailed in the Supporting Information Note.

We construct an NN to handle the functional output regression, cGAN, which consists of a generative model called a generator (*G*) and a binary classifier called a discriminator (*D*). The model structure is summarized in Figure 2 (see the Supporting Information Note for more details). With the generator *Y* = *G*(*X*,*Z*), the output vector of the *m* absorption values in eq 2 or the matrix of a grayscale microstructure image is modeled as *Y*. The input variables consist of *X* in the regression model (referred to as the conditional variable in cGAN) and random noise *Z*. The noise Z ∼ *N*(0,1) is assumed to follow a normal distribution with a mean of zero and unit variance. The input (*X*, *Z*) is first transformed into an embedding vector by passing through a fully connected embedding layer and is further transformed into a vectorized spectrum or a microstructure image *Y* = *y*(*X*), as in eq 2, by passing a series of differently stacked hidden layers depending on the task. The discriminator *D*(*X*,*Y*) is a binary classifier, in which the conditional variables *X* and *Y* are given as inputs. The discriminator judges whether object *Y* (a spectrum or an image) is real or fake. The discriminator *D* is modeled as a conventional fully connected NN or a convolutional NN.^51^ The model structure, such as the number of layers and neurons in each layer, is determined based on the generalization performance in a separate validation data set, while maintaining the basic form described here. The detailed settings for each problem are described in the Supporting Information Note.

With this composite modeling, *G* and *D* are trained alternatively according to the following minmax strategy
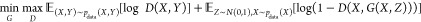
3

The first term becomes larger as *D*(*X*,*Y*) increases, that is, when the discriminator *D* correctly identifies the input real object as real. The second term becomes large when 1 – *D*(*X*,*G*(*X*,*Z*)) becomes large, that is, when *D* successfully recognizes the fake *Y* = *G*(*X*,*Z*) to be fake. The discriminator *D* is learned such that the classification error is minimized, and *G* is trained to reduce the second term such that *D* is misrecognized. By alternately training *G* and *D*, we derive *G*, which can produce high-quality fake spectral functions or microstructure images for any given descriptor *X*. For the spectral prediction, we generate *r* random samples {*Z*_*i*_|*i* = 1, ..., *r*} (*r* = 100) and use the ensemble  of the learned generator to improve the smoothness of the predicted function.

cGAN can be regarded as a supervised learning technique for multidimensional output variables. Because cGANs have been intensively studied, particularly for image-generation tasks, when treating images as the output variable, we can take advantage of the wealth of tips and various extended works that have been accumulated in machine-learning research. However, like other conventional generative adversarial networks, cGANs suffer from instability during the learning process. In particular, vulnerability to small data sets has been pointed out in many previous studies. With limited amounts of training data, the discriminator can easily overfit the data to make a perfect true/false classification, which leads to gradient vanishing and halting of the learning process before a sufficiently accurate generator (the target regression model) is created. The solution to stabilize the adversarial training process is to balance the learning progress of *G* and *D*, but this is not an easy task. The primary reason for this is the over-parameterization of the generator caused by the high dimensionality of the vectorized object *Y*. In the case studies shown below, the dimension of *Y* is more than 170 or 2000 for spectral function prediction and up to 10,000 for microstructural microscope images with a resolution of 100 × 100. For example, to describe the mapping from *X* to the high-dimensional image object *Y*, it is necessary to introduce one or more transposed convolution matrices of large sizes. In applications shown later, the total numbers of model parameters in the trained generators reached the order of 2.7 million or 10 million for the spectral function prediction and 5.8 million for microstructure image prediction.

### Kernel Regression with Functional Outputs

In addition to cGAN, we present another model with a simple, naturally interpretable model structure. The design concept is inspired by regression models for functional outputs, which have been studied in the context of functional data analysis. The functional output *Y*(*X*,*t*) is modeled as follows
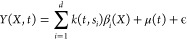
4

The first term is the weighted sum of the *d* kernel basis functions {*k*(*t*,*s*_*i*_)|*i* = 1, ..., *d*}. The kernel centers *s*_*i*_ are equally spaced in the domain of wavelengths or image coordinates. In this study, we use the Gaussian radial basis function (RBF) kernel as
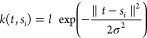
5

The variance σ^2^ > 0 and length scale *l* > 0 are hyperparameters adjusted based on the evaluation of the generalization performance. Alternatively, one may predetermine the hyperparameters based on the empirically known resolution of a measurement system or the inherent variation of *Y*(*X*,*t*) in varying *t* for the physical system of interest. The regression coefficient β_*i*_(*X*) depends only on the input variable *X*, which is modeled by a NN, as described below. μ(*t*) is a baseline function estimated by imposing smoothness as a regularizer. ϵ denotes the noise term.

The overall model represents a system in which each kernel function pre-arranged in the domain of variable *t* is activated or deactivated depending on the input variable *X*, for example, the presence or absence of a specific fragment in a fingerprinted chemical structure *X*. One advantage of this modeling is that the number of parameters can be reduced by controlling the number of kernels *d* placed in the domain *t*. In the cGAN generator, the number of neurons in the output layer inevitably increases because it is constrained by the dimensionality of *Y*.

**Figure 3:**
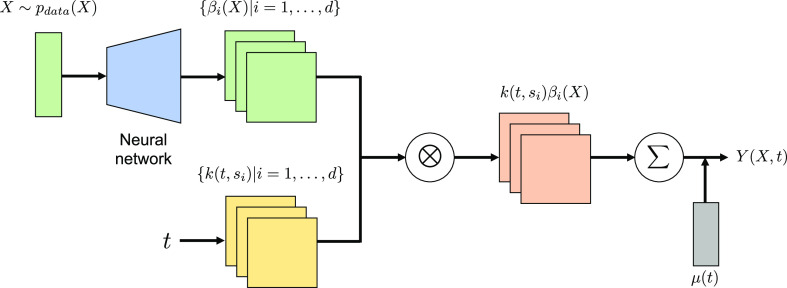
Model architecture of the functional output kernel regression. The coefficient functions {β_*i*_(*X*)|*i* = 1, ..., *d*} for a set of *d* pre-defined kernels {*k*(*t*,*s*_*i*_)|*i* = 1, ..., *d*} depend only on input *X*. The mapping from *X* to β_*i*_(*X*) is modeled by a fully connected or convolutional NN. The number *d* of kernel functions is appropriately controlled to be smaller than the number of observations for *t*, that is, *d* ≤ *m*.

The regression coefficients {β_*i*_(*X*)|*i* = 1, ..., *d*} are modeled differently using NNs for the two tasks (Figure 3, Supporting Information Note). We use NNs with a structure similar to that of the generator in cGAN. In the prediction of the optical absorption spectra, the input *X* is given by a 1024 binary vector that encodes the chemical structure of a molecule using the extended connectivity fingerprint^10^ with a radius of 3. The mapping from *X* to the *d* output coefficients is modeled by multiple blocks of stacked hidden layers, including a fully connected layer, batch normalization layer, and leaky ReLU activation function.^52,53^ For the microstructure image prediction, as detailed later, the input *X* includes six processing parameters and a Gaussian noise. The mapping from *X* to the *d* output coefficients consists of multiple blocks of stacked layers, including a transposed conventional layer, batch normalization layer, and leaky ReLU activation function. Hyperparameters such as the number of convolutional layers and neurons are tuned based on a separate validation data set. See the Supporting Information Note for the procedure for hyperparameter tuning.

In model training, the following objective function is minimized with respect to the parameters in the model of {β_*i*_(*X*)|*i* = 1, ..., *d*} and the baseline function μ(*t*)
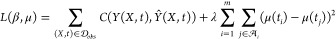
6

The first term involves the discrepancy *C* between an observed *Y*(*X*,*t*) and its prediction counterpart . In the experiments reported later, for both spectral prediction and microstructure prediction, *C* is defined by an ordinary squared loss, which is summed over all observations of *X* and *t*. The second term is the regularization term for the baseline function. Regularization induces a smooth transition between μ(*t*_*i*_) and μ(*t*_*j*_) for an observation point *t*_*i*_ and its neighborhood . In this formulation, the observed series of *Y*(*X*,*t*) is allowed to have some missing values for *t*.

## Results and Discussion

We highlight the potential prediction capability and learning mechanism of the proposed methods by presenting three application examples. The objective of the first two examples is to predict the function of the UV–vis or NIR absorption spectrum of an organic molecule, taking its chemical structure as the input. In the first case, the number of samples was approximately 2200 or less, whereas in the second case, the number of samples was only 68. The objective of the third example is to predict electron microscopy images of microstructures from the composition and processing conditions of the fabricated thin metal films.

### Prediction of UV–Vis Absorption Spectrum

Urbina et al.^31^ produced two experimental data sets of UV–vis spectra that encompass different chemical spaces. In the paper, these data sets were referred to as Dataset I and Dataset II. Dataset I consists of the absorption spectra of 949 different commercial compounds measured using a high-performance liquid chromatography system. Dataset II contains the spectra of 2222 different commercially available pharmaceutical molecules, which were measured with a spectrophotometer in a multi-well plate format. The absorption spectra were measured at 181 and 171 wavelengths equally spaced in the UV–viz (220–400 nm) for Dataset I and Dataset II, respectively. Compared to Dataset I, Dataset II has a larger amount of data and a larger diversity of chemical structures. For model training and evaluation of prediction performance, approximately 70% of the compound set was randomly selected as the training set and the remaining approximately 15% as the test set. Partitioning of the data set was performed according to the compound species to avoid multiple spectral profiles of the same compound leaking into the test set. In Urbina et al.,^31^ the results of applying two encoder–decoder architectures with LSTM cells and an attention mechanism, respectively, were reported, which were compared with the performance metrics of the present methods.

For the kernel regression, the observed wavelength range 220 or 230–400 nm was divided into 128 equally spaced segments, and the kernel centers *s*_1_, ..., *s*_*d*_ (*d* = 128) were placed there. The set of hyperparameters consisted of the variance σ^2^, length scale *l* in the RBF kernel, and the number of hidden layers and neurons in the NN. For each σ^2^ and *l*, three and four grid points were set as candidates. The number of hidden layer blocks was set between 1 and 4. Once the number of blocks was determined, the number of neurons in each layer was determined, as shown in the Supporting Information Note (Figure S2 and Table S3). Correspondingly, the total number of candidates for the hyperparameter search was 48 (=4 × 4 × 3). The generalization performance of the trained model for each candidate was measured using the root mean square error (RMSE) on the validation data set, and the best combination was identified.

The hyperparameter of cGAN was given by the network structure of the generator and discriminator. As in the kernel regression, both network structures consisted of a series of stacked hidden layers, with each block consisting of a fully connected layer, a batch normalization layer, and a leaky ReLU activation function. The difference from the kernel regression was that the output variables were directly formed by the *m* absorbances of the different wavelengths in the spectral function. The number of blocks to be searched was between 1 and 4, and the number of neurons in each layer was determined, as shown in the Supporting Information Note (Figure S1 and Table S1). The generalization performance of the models was measured using the RMSE of the validation data set to identify the best hyperparameter combination.

Through validation, in Dataset I, the numbers of stacked hidden layers for the NNs in the kernel regression, generator, and discriminator of cGAN were selected as 4, 4, and 2, respectively. In Dataset II, the number of stacked hidden layers for the NNs in the kernel regression, generator, and discriminator of cGAN were selected as 3, 4, and 3, respectively. The value of σ^2^ was selected as 0.0005 for both data sets, and *l* was selected as 0.5 or 5 for Dataset I or Dataset II, respectively. To evaluate the performance of these models, we calculated the RMSE, coefficient of determination (*R*^2^), and mean absolute error (MAE) between the *m* predicted spectral values and their observations for each test molecule and compared the median of each performance metric for the 150 and 342 test molecules. We also considered the differences in the spectral series and evaluated the gradient-level prediction performance using the same procedure. These performance measures are detailed in the Supporting Information Note.

Table 1 summarizes the means and standard deviations of the performance measures for the three independent numerical experiments; the prediction accuracy of the LSTM-based and attention-based encoder–decoder models reported in Urbina et al.^31^ is also given. In Dataset I, the kernel regression outperformed the other three, but the difference with cGAN was not pronounced. The reason for the low prediction accuracy of the difference spectrum of cGAN is due to the use of randomly sampled *Z* in the calculation of the predicted spectra, which resulted in a loss of smoothness in the gradient of the spectrum. However, this problem can be solved by smoothing the predicted spectrum in the post-processing step. As the code for the encoder–decoder models is not distributed, we could not go further into the comparison of the models, but it should be concluded that in this case study, they have almost the same performance.

**Table 1:** Comparison of the Prediction Accuracy for Optical Absorption Spectral Functions^a^

		RMSE	*R*^2^	MAE	RMSE derivative
Dataset I	LSTM	0.169 ± 0.132	0.626 ± 1.166	0.119 ± 0.106	0.013 ± 0.010
	attention	0.154 ± 0.144	0.680 ± 1.230	0.091 ± 0.120	0.018 ± 0.020
	kernel	**0.111** ± **0.009**	**0.798** ± **0.043**	0.075 ± 0.006	**0.012** ± **0.001**
	cGAN	0.112 ± 0.014	0.786 ± 0.057	**0.071** ± **0.010**	0.053 ± 0.002
Dataset II	LSTM	0.064 ± 0.062	0.710 ± 0.472	0.047 ± 0.075	0.008 ± 0.006
	attention	**0.055** ± **0.071**	0.699 ± 0.259	**0.044** ± **0.052**	**0.006** ± **0.007**
	kernel	0.093 ± 0.004	0.655 ± 0.007	0.066 ± 0.001	0.009 ± 0.000
	cGAN	0.085 ± 0.002	**0.718** ± **0.022**	0.058 ± 0.003	0.016 ± 0.001
USGS	kernel	**0.076** ± **0.024**	**0.602** ± **0.200**	**0.053** ± **0.009**	**0.022** ± **0.003**
	cGAN	0.074 ± 0.003	0.493 ± 0.102	0.058 ± 0.009	0.528 ± 0.043

a Dataset I and Dataset II cover the UV–vis spectra, and the USGS spectral library covers data in the NIR domain. The performance metrics shown for the LSTM and attention-based encoder–decoder models are those reported in Urbina et al.^31^

**Figure 4:**
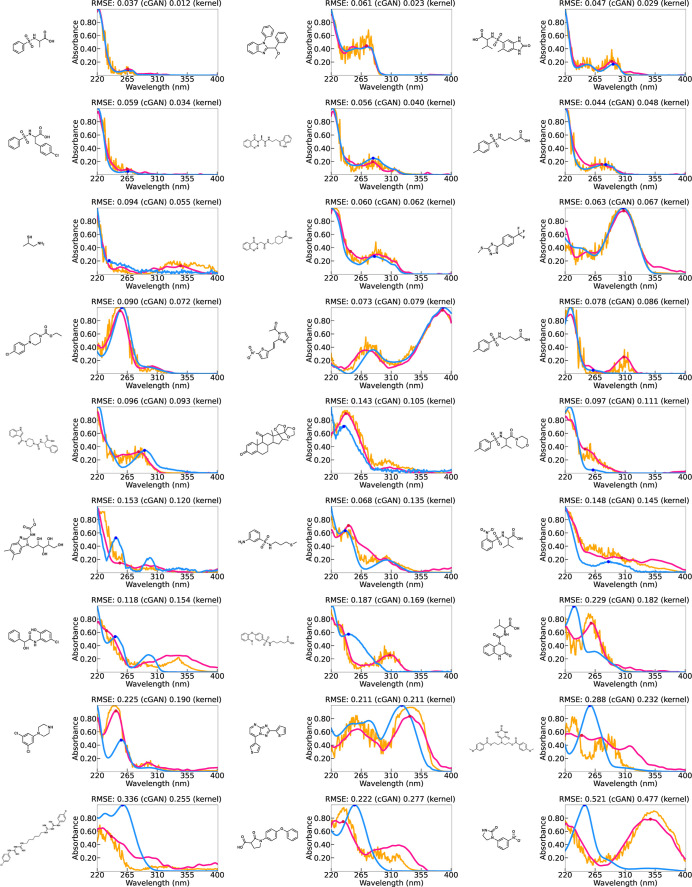
Prediction results of (a) the functional output kernel regression (pink) and (b) cGAN (orange) with observed UV–vis spectra (blue) in Dataset I.

**Figure 5:**
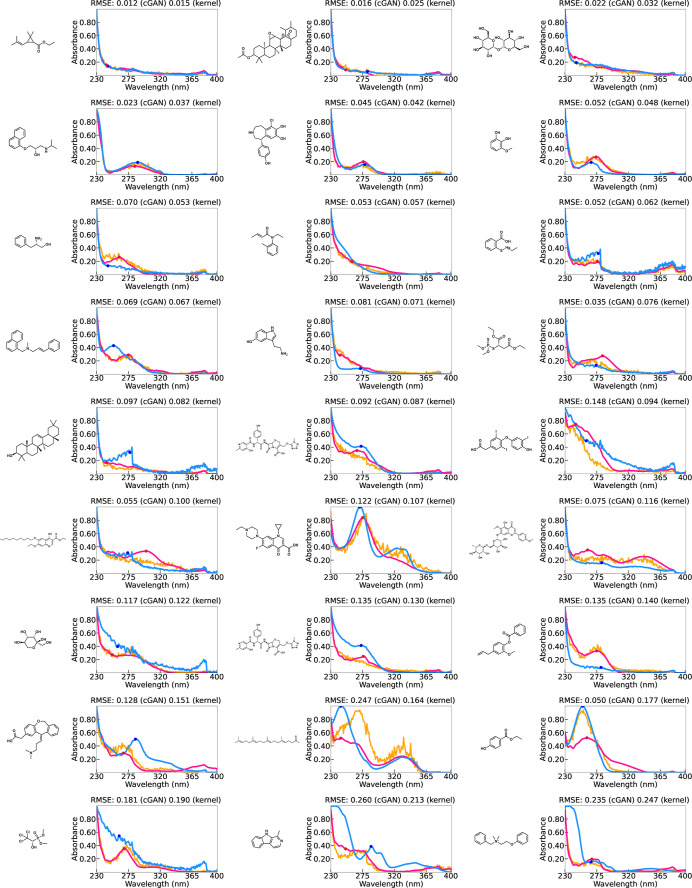
Prediction results of (a) the functional output kernel regression (pink) and (b) cGAN (orange) with observed UV–vis spectra (blue) in Dataset II.

Here, the predicted spectral functions and their observed values are presented exhaustively to obtain a view of the predictive capability of the kernel regression and cGAN. Figures 4 and 5 show the prediction outcomes for 27 randomly selected test molecules in Dataset I and Dataset II, respectively. To obtain a more comprehensive view of the prediction accuracy, Supporting Information Note (Figures S3 and S5) also provides the results of 60 randomly selected test molecules in each data set (see also Figures S4 and S6 for the results of training). According to a careful visual inspection, it can be seen that the models retain a surprisingly high prediction accuracy. For a significant number of molecules, the positions of multiple peaks in the absorption spectrum and the shape of the function were almost perfectly predicted. In some cases, even features that are not visually noticeable, such as plateaus or tiny peaks, were captured appropriately. This observation suggests that the presence or absence of chemical substructures is a major factor in the optical absorption spectra of molecules. There was no significant difference between the kernel regression and cGAN in terms of capturing the broad trend of the spectral function. However, as mentioned above, cGAN requires some effort to detect the peak position because of the noise fluctuations of *Z* in the prediction equation. Even when smoothing is applied, unexpected false peaks can occur. Therefore, we conclude that the kernel regression has an advantage in terms of the spectral prediction.

### Spectral Prediction with Limited Data in the USGS Spectral Library

The U.S. Geological Survey (USGS) Spectral Library Version 7^54^ contains reflectance optical spectra of over 1000 different molecules, each of which was measured in three different sets of environments: laboratory, field, and airborne spectrometers. Of these samples, 68 organic compounds that were measured in the laboratory at room temperature were pre-extracted. The compiled data set contained no case where multiple spectra were assigned to the same compound. All spectral values were recorded at 2151 common discrete points approximately equally spaced in the wavelength range 0.35–2.5 μm from the ultraviolet to the far infrared. Compared to the number of UV–vis data set shown above, the sample size of 68 was quite small. Note that when representing a function using a sum of RBF kernels, the shape of the function to be predicted is required to be smooth. However, the reflectance spectra of the original data showed multiple sharp downward spikes, making it difficult to represent them as a weighted sum of RBFs. Therefore, a smooth spectral function was defined as the output variable using the conversion equation *A* = log_10_ 100/*R* from reflectance *R* to absorbance *A*.

The model structure and procedure for selecting hyperparameters for the cGAN and kernel regression were exactly the same as those for the analysis of the UV–vis spectra described above. Randomly selected instances of 80, 10, and 10% from the entire data set were used for model training, hyperparameter validation, and performance evaluation, respectively. In addition, data splitting was independently performed three times to calculate the mean and variability of the performance measures. The numbers of stacked hidden layers for the NNs in the kernel regression, generator, and discriminator of cGAN were selected as 3, 3, and 2, respectively. The values of σ^2^ and *l* were selected as 0.0005 and 1, respectively.

**Figure 6:**
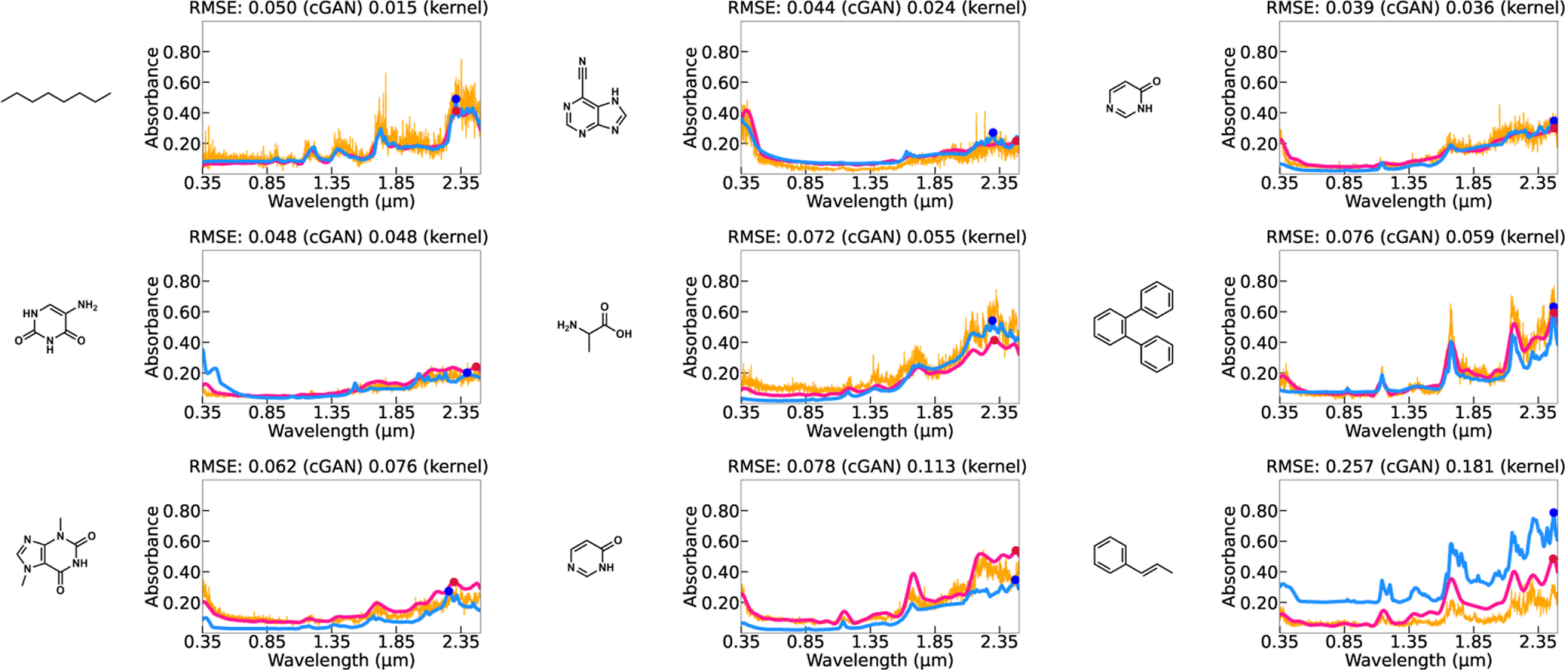
Prediction results of (a) the kernel regression (pink) and (b) cGAN (orange) for the optical absorbance spectra of nine test molecules with experimental profiles (blue) in the USGS spectral library.

The prediction accuracy of cGAN and kernel regression for the test instances, which were trained on the optimized hyperparameters, are summarized in Table 1 (see the Supporting Information Note for details on the performance measures). Unlike the results of the UV–vis spectra, the kernel regression overwhelmingly outperformed cGAN in accuracy. For example, the *R*^2^ values for cGAN and kernel regression were 0.493 ± 0.102 and 0.602 ± 0.200, respectively, and the MAEs were 0.058 ± 0.009 and 0.053 ± 0.009, respectively. It is likely that for the given small data set, the over-parameterization of the generator in cGAN caused degradation in the predictive performance such as getting stuck in a poor local optimum. However, the kernel regression reached a sufficiently high prediction accuracy despite being trained on only 54 samples. Figure 6 shows the predicted and true spectra for several cases (see Figure S7 for a more comprehensive visualization of the prediction results and Figure S8 for the result of fitting to the training data). As in the UV–vis cases, the peak positions and functional features of the observed spectra can generally be predicted. We also observed that, in many cases, the plateau and minor peaks could be properly captured. The kernel regression has a strong tolerance for a limited sample size, which will be further investigated later.

### Microstructure Image Prediction

A thin-film material consisting of a chromium (Cr)-based metal plate coated with aluminum (Al) was analyzed.^34^ Cr and Al metal plates were placed face-to-face, and a mixed gas of nitrogen (N) and argon (Ar) was sprayed onto the Al plate at high speed by magnetron sputtering, so that the ejected Al atoms were adsorbed onto the Cr plate. Under high-temperature conditions, the metal plates were contaminated with oxygen (O) from residual gas outgassing from the deposition equipment. The input  to the model is a six-dimensional real vector representing the composition Cr_1–*x*_Al_*x*_O_*y*_N and the processing conditions.

1. Cr and Al content *x*
2. O content *y*
3. Temperature at which Al is adsorbed (*T*_*d*_)
4. Pressure at which Al is adsorbed (*P*_*d*_)
5. Average energy of the Ar ions when they are incident on the Al metal plate (*E*_*i*_)
6. Ionization degree of the Ar gas (*I*_*d*_)

Each element of the input vector *X* was normalized to have a mean of zero and a unit variance in the training data. The output variable *Y* is the SEM image of the microstructure with a resolution of 100 × 100.

In the functional output kernel regression, the centers of 32 × 32 RBF kernels (*d* = 32 × 32) were placed at equally spaced positions in the two-dimensional image coordinate space. The modeling and training conditions were almost the same as those used to predict the spectral functions. The hyperparameter set to be explored was given by the network structure and the variance and length scale of the RBF kernel. The input variable was transferred to the embedding latent space via a fully connected layer. A repeating unit consisting of a transpose convolution layer and a leaky ReLU activation function was applied several times to this embedding vector. Here, the basic structure of the model was the same as that in the spectral function prediction, but a Gaussian noise was augmented to the input system, as in cGAN, to increase the diversity of representable image patterns as detailed in the Supporting Information Note. The cGAN modeling was also similar to that in the spectral function prediction. The input variable was transformed into the output variable via a series of fully connected layers to obtain a feature embedding, a repeated application of the transpose convolution layer and leaky ReLU to the embedding feature, and a fully connected layer to generate a 100 × 100 image *Y* (Supporting Information Note).

The data contained 123 SEM images with their compositional and processing conditions, of which 90, 5, and 5% were randomly assigned to the training, validation, and test sets, respectively. The data partitioning was independently repeated thrice. The generalization performance of the model was investigated by varying the number of layers from 1 to 4. The number of neurons in each layer was designed as described in the Supporting Information Note. The numbers of stacked hidden layers for the NNs in the kernel regression, generator, and discriminator of cGAN were selected as 4, 1, and 2, respectively. The values of σ^2^ and *l* were selected as 0.0001 and 5, respectively.

**Figure 7:**
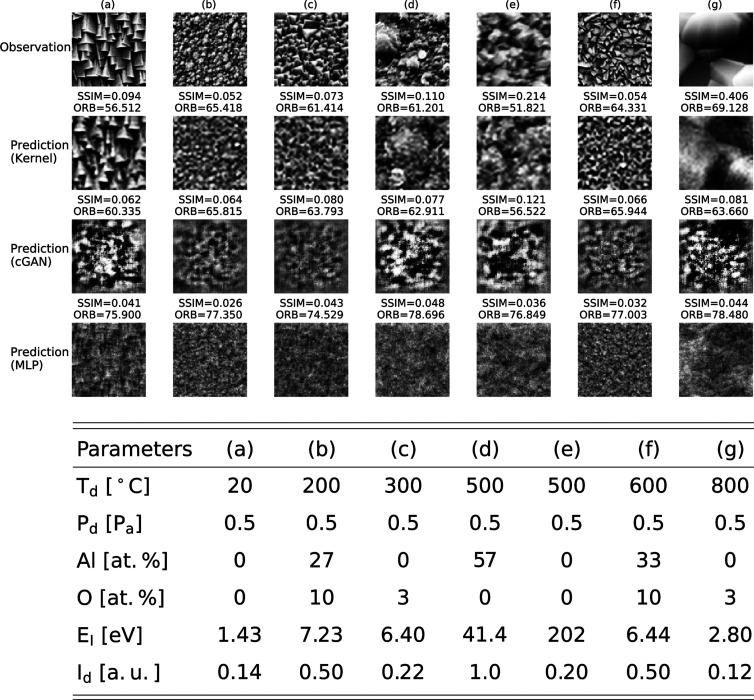
Results of the microstructure prediction using the functional output kernel regression and cGAN and pixel-by-pixel prediction using a MLP with respect to seven different SEM images.

**Figure 8:**
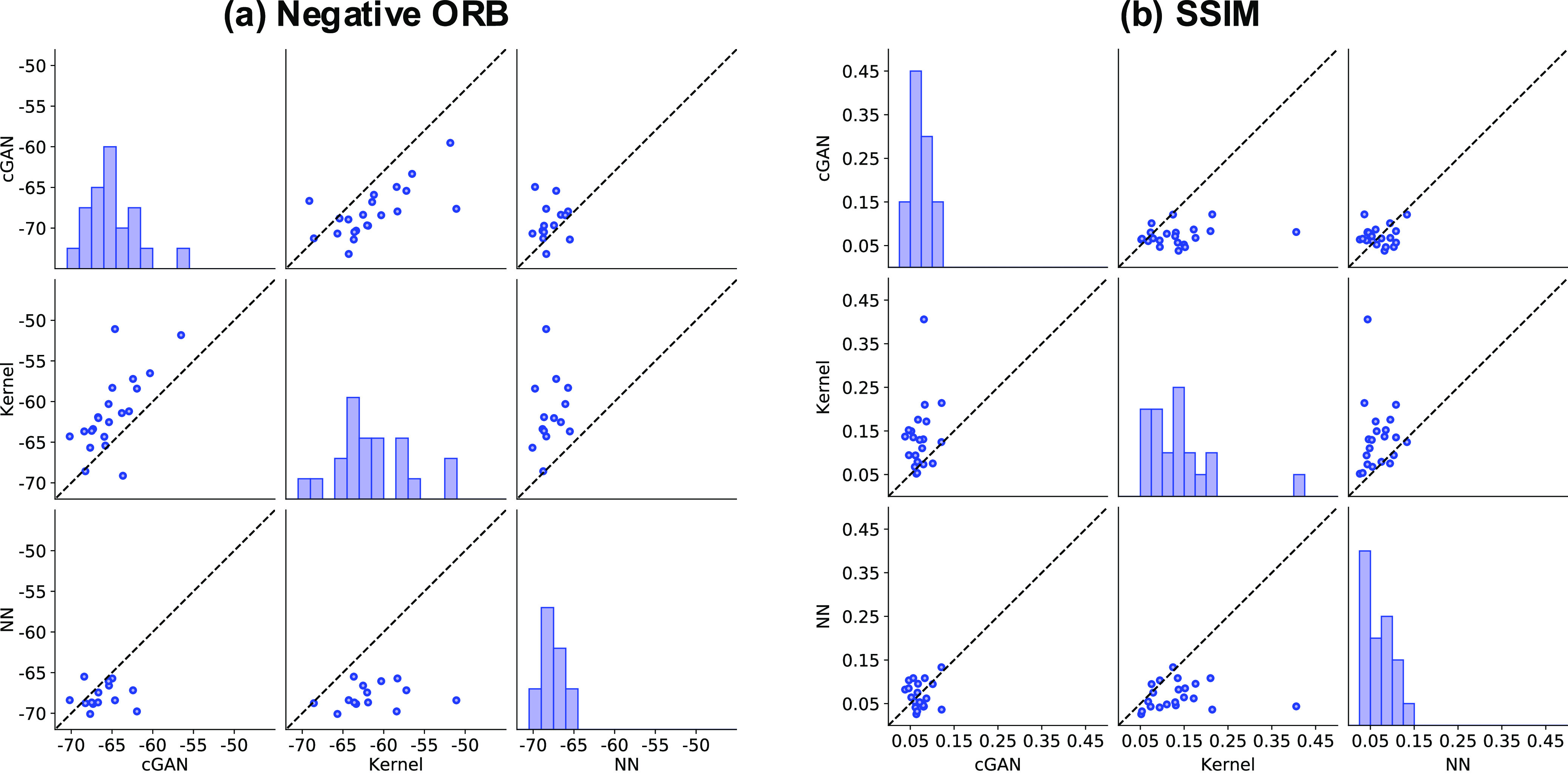
Scatter plot matrices showing the distribution of two similarity measures (the ORB with negative transformation and SSIM) of the predicted SEM images with respect to test data for cGAN, functional output kernel regression, and pixel-by-pixel independent prediction using conventional NNs.

Figure 7 shows the experimental and predicted SEM images for the functional output kernel regression and cGAN for seven randomly selected test conditions. To obtain a comprehensive view of the prediction performance, the Supporting Information Note provides the prediction results for 14 randomly selected test conditions and training results for 60 randomly selected conditions. Comparing with the experimental SEM images, the predicted images of the functional output kernel regression properly captured the difference in morphological features of microstructures, such as grain sizes, even though the amount of training data was as small. The cGAN model was unable to predict observed features of microstructures, possibly due to the limited amount of training data. We calculated the negative-transformed oriented FAST and rotated BRIEF (ORB)^55^ and structural similarity (SSIM)^56^ as measures of image similarity invariant to shifts in position, scale, and rotation (Supporting Information Note). Figure 8 summarizes the comparison of these two measures for the functional output kernel regression and cGAN with respect to the 21 test instances from the three independent trials. Clearly, the functional output kernel regression outperformed cGAN in both similarity measures.

As with the spectral prediction, a high learning potential of the function output kernel regression on small data was confirmed. However, the experimental results also indicated the weakness of the trained model as an image generator. Although the predicted images successfully captured the morphological features of the microstructures, they were less clear than those of the experimental images. The reduction in sharpness was caused by the blurring effect of using kernel functions. For functional output regression, the parameter savings using a basis set of kernel functions leads to high tolerance for small data, but at the same time, it inevitably leads to a reduction in the quality of the generated images. However, unlike image generation in computer vision, the generation of high-quality images is not as important for prediction tasks in materials science. If sharpness or contrast needs to be increased, various techniques of image synthesis can be employed. Besides the blurring effect caused by using the kernel functions, the use of the squared loss of per-pixel image intensity would induce a reduction in the sharpness of generated images. To enhance sharpness and contrast, the gradient image similarity between the generated and real images could be added to the loss function, that is, sharpness loss regularization.^57,58^ Furthermore, replacing the squared loss with -norm loss, as in pix2pix-GAN,^59^ a well-known generative model for image-to-image translation, would reduce blurring.

### Tolerance to Data Size in Functional Output Kernel Regression

We investigated the underlying mechanisms behind the learning success despite the exceedingly small amount of data, that is, 54 training samples for spectral prediction and 100 or more for microstructure prediction. The functional output regression has a learning mechanism that is common to multi-task learning. The tasks of predicting multiple function values are not independent but are related to each other. Joint learning of multiple related tasks is expected to be advantageous for the model to acquire common representations across tasks. In addition, data accumulation by the simultaneous use of data from multiple tasks is generally advantageous in suppressing overlearning of task-specific noises. These mechanisms may be responsible for the success in building the model with a small amount of data. To confirm this hypothesis, we conducted three numerical experiments using the optical absorption spectra in Dataset I.

(a) The maximum absorption wavelength λ_max_ and its intensity value were calculated from the spectral function predicted by the functional output kernel regression, and the accuracy of predicting λ_max_ and the maximum intensity were verified against their observed values.
(b) Using λ_max_ and its intensities as a data set extracted from the observed spectra in advance, we built a multi-layer perceptron (MLP) NN that directly predicts the extracted scalar values of λ_max_ or the maximum intensity from the fingerprinted chemical structure (the same as the one used in the cGAN and kernel regression).
(c) We trained *m* MLPs independently and separately to predict the spectral values of each of the *m* equally spaced grid points. The estimated λ_max_ and the maximum intensity values were calculated from the pointwise prediction of the spectral function.

In the peak detection from a spectral profile, λ_max_ was selected as the wavelength of the maximum intensity among the maxima that appeared 232 nm from the left end. In these three experiments, two different ratios of the training, validation, and test sets were set to 649:151:150 and 100:425:425, respectively. The data partitioning was repeated 10 times independently, and training and testing were performed. For the evaluation of the accuracy of the test set, cases in which the predicted peak position was within 10 nm of the observed peak position were determined as correct and cases in which the predicted peak intensity was within 0.1 of the observed intensity were determined as correct. As shown in Table 2, for both models obtained from the smaller and larger data sets, the prediction accuracy of the functional output kernel regression with the entire function trained simultaneously (model (a)) overwhelmingly exceeded the performance of models (b) and (c). In particular, compared to model (c), which learned and predicted spectral values independently, model (a) showed little degradation in prediction performance when the amount of training data was reduced to 100.

**Table 2:** Tolerance to Small Data in Predicting the Maximum Absorption Wavelength λ_max_ and Peak Intensity^a^

method	data split	position λ_max_ (%)	intensity of λ_max_ (%)
(a) kernel	649:151:150	46.3	43.9
(b) MLP for λ_max_		23.5	11.7
(c) MLP for each wavelength		45.9	45.5
(a) kernel	100:425:425	36.1	32.6
(b) MLP for λ_max_		22.4	11.1
(c) MLP for each wavelength		32.9	25.5

a Three cases were tested: (a) feature values obtained from the predicted spectral function of the functional output kernel regression, (b) the direct prediction of the two feature quantities using MLPs, and (c) feature values obtained from a set of MLPs with pointwise learning and prediction. The ratios of the training, validation, and test sets were set to 649:151:150 and 100:425:425, respectively.

We performed a similar test with the microstructure image prediction. The performance of the functional output kernel regression was compared to that of the pixelwise prediction by MLPs that learned the intensity of each pixel in the image independently. As illustrated in Figure 7, the pixelwise MLPs clearly failed to predict any test instances. Figure 8 shows that the similarity values of the predicted images from the pixelwise learning to the 21 test images were significantly lower than those of the functional output kernel regression in all cases.

This observation has implications for the methodological construction of machine learning-based material-property prediction. For example, in the prediction of a temperature-dependent property, it is often the case that the temperature range is limited to room temperature to define a target property to be predicted. However, such an approach makes the problem more difficult and reduces prediction accuracy. In fact, the pixel-by-pixel machine learning failed to predict the image intensity at all, but successfully predicted the entire images with a fairly high degree of accuracy. When functional data are available, direct prediction of the functional output variable can significantly improve prediction accuracy.

## Conclusions

In the past few years, several machine learning techniques have been introduced in material science. In this context, machine learning techniques for predicting the physicochemical values of scalar quantities from input materials have matured. On the other hand, in this study, we focused on the regression problem where the output variable is in the form of a function. We described the potential problem setting in material science and presented two methodologies based on deep generative models and statistical functional data analysis. Because machine learning research based on this perspective is still in its infancy, there must be other promising methods besides the one proposed here. As a starting point, we demonstrated the potential of functional output regression in two cases: the prediction of the light absorption spectral function of a molecular system and the prediction of the microstructure from experimental condition parameters.

Of particular interest is the mechanism of the high tolerance of the functional output regression to the limited amount of training data. In this study, the amount of data was much smaller than in general situations: 54 samples in the prediction of absorption spectra and 109 samples for microstructure image prediction. Despite the extremely small sample sizes, we successfully obtained models with sufficiently high prediction accuracies. In general, machine learning prediction of physical or chemical properties is often performed by transforming the functional data into a few features and then predicting the scalar output variables, instead of directly predicting the functional data. However, the experimental results suggest that the direct prediction of functional output variables may involve a learning mechanism that favors the acquisition of higher generalization performance than the prediction of scalar variables. Intuitively, the higher dimensionality of the output variable is likely to lead to overlearning and a decrease in the generalization performance of the trained model. However, because the tasks of simultaneously predicting multiple function values are not independent but strongly related to each other, the simultaneous use of data from multiple tasks can suppress task-specific noise due to data expansion. In addition, in multi-task modeling, the complexity of the model does not increase significantly as usually only one- or two-dimensional input variables *t* are added. In the trade-off between increasing model complexity and data expansion, the advantages of the latter tend to outweigh those of the former.

There are many other potential applications in material research where the prediction of functional outputs is required. Most material properties are given as functions of temperature or frequency. The dielectric properties are defined as functions of temperature and frequency. Polymeric properties such as the specific volume, coefficient of linear expansion, bulk modulus, specific heat, thermal conductivity, and χ parameters are also determined in a temperature-dependent manner. From these temperature-dependent curves, important properties such as the glass transition temperature, crystal melting point, and crystallization temperature can be calculated. The dependence of properties on processing conditional parameters is another typical application of functional output regression. Furthermore, in the 2D and 3D imaging of material structures, the output variable is given as a multidimensional function in the image coordinate space. Due to the lack of data availability, we have presented only a few limited applications, but there are many problem settings for functional output regression that remain unexplored in material research. In principle, the proposed methods are designed to handle arbitrary high-dimensional functional data. We hope that the distributed Python code can be utilized to discover more problem settings.

## Data and Software Availability

The Python codes for the functional output kernel regression and pretrained models are available on GitHub.^41^ Optical absorption spectra are available from Urbina et al.^31^ for Dataset I and Dataset II and Kokaly et al.^54^ for the USGS library. SEM images of the microstructures with compositional and processing data are available from GitHub https://github.com/lbanko/generative-structure-zone-diagrams provided by Banko et al.^34^

## Supporting Information Available

Modeling details for cGAN and the functional output kernel regression, procedure of hyperparameter search, results of predicting the optical absorption spectra in the UV–vis region and in the USGS spectral library, and the microstructure image prediction (PDF)
