# Supplementary material for: Functional Output Regression for Machine Learning in Materials Science
Source: J Chem Inf Model. 2022 Oct 10;62(20):4837–51. doi: 10.1021/acs.jcim.2c00626 (PMC9597664; doi:10.1021/acs.jcim.2c00626)
Supplement: Supplementary file 1 — ci2c00626_si_001.pdf [file ci2c00626_si_001.pdf]

Supplementary Note
Functional Output Regression for Machine Learning in Materials Science

Megumi Iwayama,[†,‡] Stephen Wu,[†,¶] Chang Liu,[¶] and Ryo Yoshida[*,†,¶,§]

†*The Graduate University for Advanced Studies, Department of Statistical Science, Tachikawa, 190-8562, Japan*

‡*Production Management Headquarters, Process Technology Division, Daicel Corporation, Himeji, 671-1283, Japan*

¶*Research Organization of Information and Systems, The Institute of Statistical Mathematics, Tachikawa, 190-8562, Japan*

§*National Institute for Materials Science, Research and Service Division of Materials Data and Integrated System, Tsukuba, 305-0047, Japan*

E-mail: yoshidar@ism.ac.jp

Contents

Conditional GAN **3**

Functional output kernel regression **5**

Performance metrics **8**

Prediction of UV-Vis absorption spectra **10**

Prediction of absorption spectra in the USGS spectral library **11**

Prediction of microstructure SEM images **11**

List of Figures

S1 Network design of candidate models for the cGAN discriminator and generator in the prediction of the spectral function ((a) and (b)), and in microstructure image prediction ((c) and (d)). 6

S2 Network design of candidate models for the kernel regression (a) in the prediction of the spectral function and (b) in the microstructure. 8

S3 Dataset I: Predicted absorption spectra of the trained cGAN (orange) and functional output kernel regression (pink) for 60 randomly selected test molecules in Dataset I with their experimental spectral profiles (blue). 16

S4 Dataset I: Results of fitting to training data for cGAN (orange) and functional output kernel regression (pink) with their experimental spectral profiles (blue). 17

S5 Dataset II: Predicted absorption spectra of the trained cGAN (orange) and functional output kernel regression (pink) for 60 randomly selected test molecules in Dataset II with their experimental spectral profiles (blue). 18

S6 Dataset II: Results of fitting to training data for cGAN (orange) and functional output kernel regression (pink) with their experimental spectral profiles (blue). 19

S7 USGS spectral library: Predicted absorption spectra of the trained cGAN (orange) and functional output kernel regression (pink) for 21 test molecules in the library with their experimental spectral profiles (blue). 20

S8 USGS spectral library: Results of fitting to training data for cGAN (orange) and functional output kernel regression (pink) with their experimental spectral profiles (blue). 21

S9 Microstructure prediction: Predicted images of the trained cGAN, functional output kernel regression and MLPs, are shown for 14 test instances with their experimental SEM images. 22

S10 Reproduction of training data: Reproduced images of trained functional output kernel regression are shown for 60 cases with their experimental SEM images. 23

List of Tables

S1 Candidate models for cGAN (discriminator and generator) in the prediction of optical absorption spectra. Asterisks denote the best-performing model selected through model validation. 13

S2 Candidate models for cGAN (discriminator and generator) in the microstructure prediction. Asterisks denote the best-performing model selected through model validation. 14

S3 Candidate models for functional output regression in the prediction of optical absorption spectra. Asterisks denote the best-performing model selected through model validation. 14

S4 Candidate models for functional output regression in the microstructure prediction. Asterisks denote the best-performing model selected through model validation. 15

Conditional GAN

Two cases are discussed in this paper: (a) prediction of the optical absorption spectrum of a molecular system and (b) prediction of the microstructure from processing and compositional conditions, but the basic form of the model is common to both. The generator describes mapping from the input variable $X \in \mathbb{R}^p$ and Gaussian noise $Z \in \mathbb{R}^q \sim N(0, 1)$ to the

output variable $Y \in \mathbb{R}^m$. The input variable is transferred to the embedding latent space via a fully connected layer, batch normalization layer, and leaky ReLU activation function. Then, a module consisting of different hidden layers depending on the task is applied several times to this embedding vector, which is finally transformed into the output variable via a fully connected layer for the two tasks. The discriminator takes a real or predicted Y and the conditional variable X as the input, and then decides whether it is true or false as the output classification probability. The binary discriminator is modeled by a fully connected neural network or a multilayer convolutional neural network, respectively, for the two tasks.

In case (a), the input variable X was a 1024-bit ($p = 1,024$) ECFP fingerprint of a chemical structure, with a radius of 3. For the generator, the dimension of the noise Z was set to $q = 100$. The generalization performance of the model was investigated by varying the number of layers and neurons in the generator and discriminator with each blocked layer consisting of a fully connected layer, batch normalization, and leaky ReLU activation function, while the number of the embedding layers and output layers was fixed to one. The number of neurons in each layer was determined by linearly interpolating the input and output lengths to a rounded integer based on the number of modules, as shown in Figure S1 and Table S1. The discriminator was modeled by an ordinary fully connected neural network. The lengths of the input spectra were 181, 171, and 2,151 for Dataset I, Dataset II, and the USGS datasets, respectively. In addition, the 1024-dimensional conditional variable X was given to the discriminator as the input. The network structure of the discriminator was designed in the same way as the generator, but the batch normalization layers were replaced with dropout layers, as shown in Figure S1.

For case (b), the designed network structures to be tested are summarized in Figure S1 and Table S2. The input variable X is a six-dimensional vector representing the chemical composition and processing conditions, and the dimension of the noise Z was set to $q = 100$. X was normalized to have a mean of zero and variance of one in the training data. For the generator, we considered four different models by varying the number of hidden layers

from one to four, with each module consisting of a transposed convolutional layer, a batch normalization layer, and a leaky ReLU activation function, where the embedding and output layers were fixed to one layer each (Figure S1). The number of neurons was determined in the same way as in case (a). The discriminator was an ordinary convolutional neural network. The size of Y was 100×100, and each value in the 6-dimensional X was copied to become a 100×100 constant matrix. Consequently, the input of the discriminator was a 7-channel 100×100 tensor. The basic network structure of the discriminator was designed in the same way as that of the generator, except that the initial embedding layer was dropped.

The set of hyperparameters to be explored was the number of hidden layers of neural networks. We varied the number of hidden layers in the generator and discriminator from one to four as in Figure S1 and Table S1 for the spectral prediction and Figure S1 and Table S2 for the microstructure image prediction, and their all possible combinations (a total of 16 cases) were considered and tested. For each of case (a) and (b), we chose a model that minimized the root mean square error (RMSE) with respect to the validation datasets.

Functional output kernel regression

The model determines a mapping from the input variable $X \in \mathbb{R}^p$ to the d regression coefficients $\{\beta_i(X) | i = 1, \ldots, d\}$. In the two cases of (a) prediction of the optical absorption spectrum of a molecular system and (b) prediction of the microstructure from processing and compositional conditions, the basic form of the model is the same. The input variables are transferred to the embedding space via a fully connected layer, batch normalization layer, and leaky ReLU activation function. A repeating module consisting of different hidden layers is applied several times to the embedding vector, which is finally transformed into the output variable via a fully connected layer or transposed convolutional layer for the two cases.

The input variable X in case (a) was a 1024-bit ($p = 1,024$) ECFP fingerprint of a chemical structure, with a radius of 3. The repeating module consisted of a fully connected

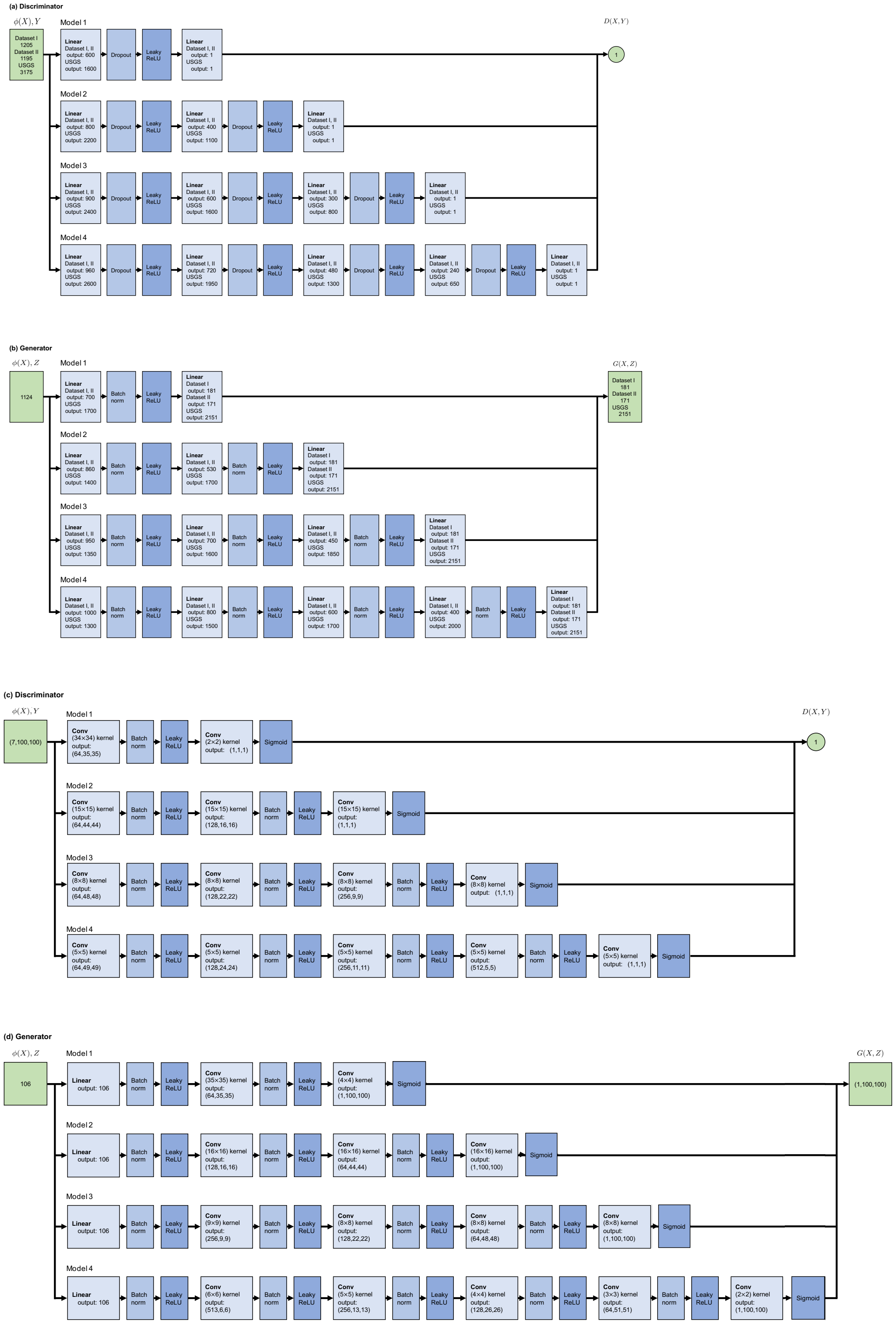

Figure S1: Network design of candidate models for the cGAN discriminator and generator in the prediction of the spectral function ((a) and (b)), and in microstructure image prediction ((c) and (d)).

layer, a batch normalization layer, and a leaky ReLU activation function. The generalization performance of the model was investigated by varying the number of modules from one to four, whereas the embedding and output layers were fixed to one layer each. The number of neurons in each layer was determined by linearly interpolating between the input and output lengths to a rounded integer based on the number of modules, as shown in Figure S2 and Table S3.

The input variable X in case (b) was a 6-dimensional vector representing the chemical composition and processing conditions, and the dimension of the noise Z was set to $q = 100$. X was normalized to have a mean of zero and variance of one in the training data. The generalization performance of the model was investigated by varying the number of modules from one to four, with each repeating module consisting of a transposed convolutional layer, a batch normalization layer, and a leaky ReLU activation function, where the embedding and output layers were fixed to one layer each. The number of neurons was determined in the same way as in case (a). Note that the 100-dimensional Gaussian random noise sampled according to $Z_i \sim N(\tau_i(X), \eta_i(X))$ $(i = 1, \ldots, 100)$ was augmented to the input system, to increase the diversity of representable image patterns (Figure S2). The mean $\tau_i(X)$ and variance $\eta_i(X)$ were modeled by using a fully connected layer from the six-dimensional input variable X. If samples from a probability distribution are included in the network, the derivative cannot be calculated in the back propagation algorithm. To solve this problem, we used the reparameterization trick, a technique widely applied in learning variational autoencoders. Using a random number $\kappa \sim N(0, 1)$ from the standard normal distribution, an element i of the random vector Z can be explicitly represented as a deterministic function of $\tau_i(X)$ and $\eta_i(X)$ as $Z_i = \tau_i(X) + \kappa\eta_i(X)$. The gradient calculations were performed based on this form.

The hyperparameter set to be explored consisted of the number of layers in the neural network, variance $l \in \{0.5, 1, 5, 10\}$, and length scale $\sigma^2 \in \{0.0005, 0.0001, 0.00005\}$ in the RBF kernel. In cases (a) and (b), the mean square error of each hyperparameter was calcu-

lated from the validation dataset, and the best combination was identified (Figure S2 and Table S3).

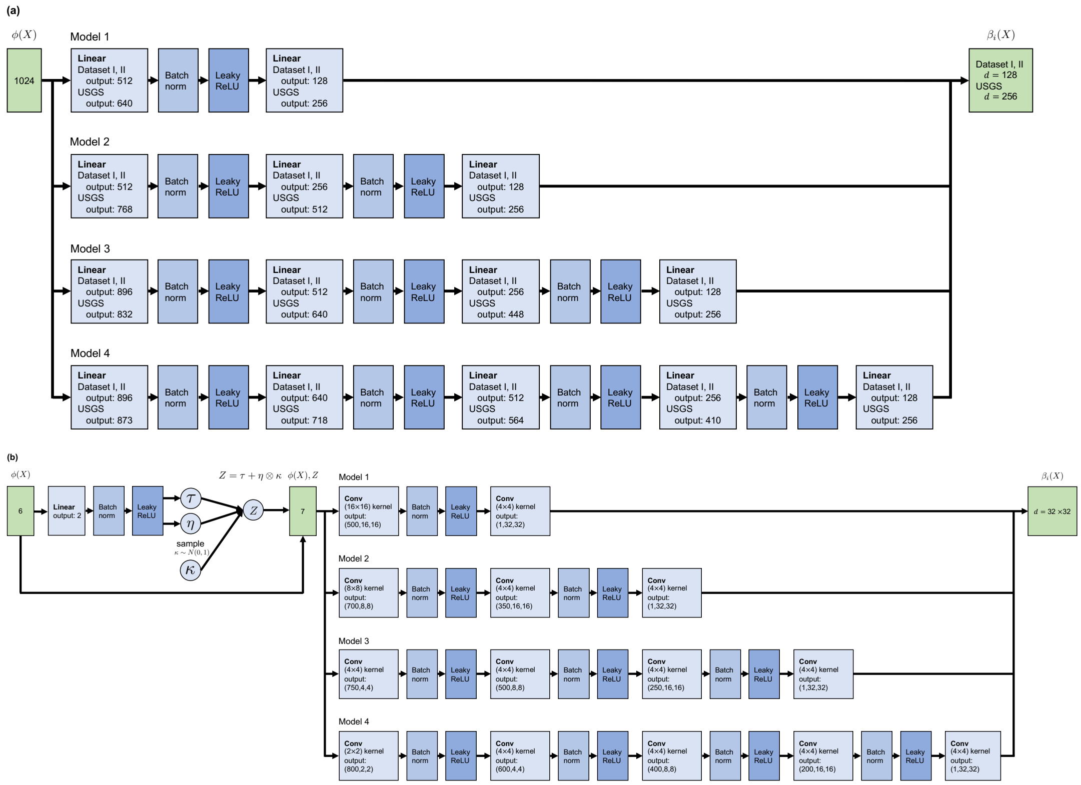

Figure S2: Network design of candidate models for the kernel regression (a) in the prediction of the spectral function and (b) in the microstructure.

Performance metrics

In the spectrum prediction where spectral profiles for n test molecules $\{X_i|i = 1, \dots, n\}$ were observed at m wavelengths $\{t_j|j = 1, \dots, m\}$, we first evaluated the predictive performance of a trained model with respect to each molecule i ($i = 1, \dots, n$) using four different metrics,

the RMSE, MAE, R^2 and RMSE of the spectral derivative, respectively, as follows:

$$
\begin{aligned}
&\mathrm{RMSE}(i) = \sqrt{\frac{1}{m}\sum_{j=1}^{m}\Big(Y(X_i,t_j) - \hat{Y}(X_i,t_j)\Big)^2}, \\
&\mathrm{MAE}(i) = \frac{1}{m}\sum_{j=1}^{m}\Big|Y(X_i,t_j) - \hat{Y}(X_i,t_j)\Big|, \\
&\mathrm{R}^2(i) = 1 - \frac{\sum_{j=1}^{m}\Big(Y(X_i,t_j) - \hat{Y}(X_i,t_j)\Big)^2}{\sum_{j=1}^{m}\big(Y(X_i,t_j) - \bar{Y}\big)^2} \quad \text{where } \bar{Y} = \frac{1}{m}\sum_{j=1}^{m}\hat{Y}(X_i,t_j), \\
&\mathrm{RMSE}_{\mathrm{derivative}}(i) = \sqrt{\frac{1}{m-1}\sum_{j=1}^{m-1}\Big[\big(Y(X_i,t_j) - Y(X_i,t_{j+1})\big) - \big(\hat{Y}(X_i,t_j) - \hat{Y}(X_i,t_{j+1})\big)\Big]^2},
\end{aligned}
\tag{1}
$$

where $\hat{Y}(X_i, t_j)$ denotes the predicted spectral value for molecule i and wavelength t_j. We then calculated the median of each metric over the n test molecules and reported their statistics across the three independent trials.

To measure the similarity between the predicted and real images, we used the oriented FAST and rotated BRIEF (ORB)[1], which holds invariance to shifts of position, scale, and rotation. ORB measures dissimilarity based on matching local feature points in two given images. The FAST algorithm[2] is used to detect the corner feature points in an image and to calculate their binary feature descriptors. It then performs feature point matching and calculates the distances between two images. To introduce scale invariance, ORB creates a multi-level image pyramid of an image and applies FAST to each layer to find feature points for every pixel. It is equal to zero when the two images are perfectly matched. See Rublee et al.[1] for details.

SSIM[3], another image similarity used in this study, compares images in terms of brightness, contrast, and structure, and calculates the similarity by multiplying them. Denoting a window of a certain size between the real o and the predicted image p as x and y, respectively,

SSIM index is calculated as follows:

$$\mathrm{SSIM}(x, y) = \frac{(2\mu_x\mu_y + c_1)(2\sigma_{xy} + c_2)}{(\mu_x^2 + \mu_y^2 + c_1)(\sigma_x^2 + \sigma_y^2 + c_2)}. \quad (2)$$

μ_x and μ_y denote the average intensities in the window of real and predicted images. σ_x and σ_y denote the standard deviations. σ_{xy} is the covariance. c_1 and c_2 are constants to stabilize the output value of SSIM when the denominator is exceedingly small. Image similarity is calculated by averaging the SSIM values as the window is moved over the image coordinates. SSIM takes values between zero and one; it is equal to one when the two images are perfectly matched.

Prediction of UV-Vis absorption spectra

The prediction results for the test instances in Datasets I and II of the UV-Vis absorption spectra were exhaustively visualized. The entire dataset was randomly divided and approximately 70%, 15%, and 15% were assigned to the training, validation, and test sets, respectively. Data partitioning was independently repeated thrice. Because it is not possible to show the prediction results for all the test cases, Figures S3 and S5 show the prediction spectra and experimental profiles of cGAN and the functional output kernel regression for 60 molecules randomly selected from Datasets I and II, respectively. Figures S4 and S6 illustrate the results of fitting of the function output kernel regression and cGAN to the training samples for Dataset I and Dataset II, respectively.

In the tolerance test for small amounts of data, we considered the prediction of the maximum absorption wavelength λ_{max} and its intensity using Dataset I. As shown in the main text of this article, three cases were tested: (a) joint learning and prediction of the entire spectral function using the functional output kernel regression, (b) direct prediction of the two scalar quantities using neural networks with multi-layer perceptron (MLP), and (c) MLPs independently and separately trained for each wavelength. In case (a) and (c),

the two features were estimated from the predicted spectrum. Two different ratios of the training, validation, and test sets were set as 649:151:150 and 100:425:425, respectively. Data partitioning was repeated ten times independently. For MLPs in case (c), we selected a network structure showing the lowest RMSE on the validation set among four candidates and a network structure showing the highest accuracy among four candidates in case (b): (1) $1024 \to 500 \to 1$, (2) $1024 \to 660 \to 330 \to 1$, (3) $1024 \to 768 \to 512 \to 256 \to 1$, and (4) $1024 \to 800 \to 600 \to 400 \to 200 \to 1$. Models (2) and (4) showed the lowest RMSEs on the validation sets in the prediction of $\lambda_{\max}$ and the maximum intensity in case (b), and model (3) was the best in case (c).

Prediction of absorption spectra in the USGS spectral library

The prediction results for the test instances from the USGS spectral library were exhaustively visualized. In total, 68 molecules were randomly split and 80%, 10%, and 10% were assigned to the training, validation, and test sets, respectively. Data partitioning was independently repeated thrice. Figure S7 shows the predicted spectra of the cGAN and functional output kernel regression for all test cases with their experimental spectral profiles. Figure S8 illustrates the results of fitting to the training samples.

Prediction of microstructure SEM images

The prediction results for the test instances in the microstructure SEM images are exhaustively visualized. In total, 123 samples were randomly split and 90%, 5%, and 5% were assigned to the training, validation, and test sets, respectively. Data partitioning was independently repeated thrice. Figure S9 shows the predicted images of the cGAN and functional output kernel regression for all the test cases with their experimental data. Figure S10 shows

60 reproduced images of the functional output kernel regression, randomly selected from the training cases and their experimental data.

In the pixel-by-pixel microstructure image prediction, we trained four MLPs with different network structures: (1) $6 \to 64 \to 1$, (2) $6 \to 64 \to 32 \to 1$, (3) $6 \to 64 \to 32 \to 16 \to 1$, and (4) $6 \to 64 \to 32 \to 16 \to 8 \to 1$. Model (4) showed the lowest RMSE on the validation set.

References

(1) Rublee, E.; Rabaud, V.; Konolige, K.; Bradski, G. ORB: an efficient alternative to SIFT or SURF. Proceedings of the IEEE International Conference on Computer Vision. Barcelona, 2011; pp 2564–2571.

(2) Rosten, E.; Drummond, T. Machine learning for high-speed corner detection. European Conference on Computer Vision. Graz, 2006; pp 430–443.

(3) Wang, Z.; Bovik, A. C.; Sheikh, H. R.; Simoncelli, E. P. Image quality assessment: from error visibility to structural similarity. *IEEE Transactions on Image Processing* **2004**, *13*, 600–612.

Table S1: Candidate models for cGAN (discriminator and generator) in the prediction of optical absorption spectra. Asterisks denote the best-performing model selected through model validation.

Category	# of layers	Dataset	Input	Layer1	Layer2	Layer3	Layer4	Output
Discriminator	1	Dataset I	1205	600				1
		Dataset II	1195	600				1
		USGS	3175	1600				1
	2	Dataset I*	1205	800	400			1
		Dataset II	1195	800	400			1
		USGS*	3175	2200	1100			1
	3	Dataset I	1205	900	600	300		1
		Dataset II*	1195	900	600	300		1
		USGS	3175	2400	1600	800		1
	4	Dataset I	1205	960	720	480	240	1
		Dataset II	1195	960	720	480	240	1
		USGS	3175	2600	1950	1300	650	1
Generator	1	Dataset I	1124	700				181
		Dataset II	1124	700				171
		USGS	1124	1700				2151
	2	Dataset I	1124	860	530			181
		Dataset II	1124	860	530			171
		USGS	1124	1400	1700			2151
	3	Dataset I	1124	950	700	450		181
		Dataset II	1124	950	700	450		171
		USGS*	1124	1350	1600	1850		2151
	4	Dataset I*	1124	1000	800	600	400	181
		Dataset II*	1124	1000	800	600	400	171
		USGS	1124	1300	1500	1700	2000	2151

Table S2: Candidate models for cGAN (discriminator and generator) in the microstructure prediction. Asterisks denote the best-performing model selected through model validation.

Category	# of layers	Input	Layer0 (Linear)	Layer1 (Conv)	Layer2 (Conv)	Layer3 (Conv)	Layer4 (Conv)	Output
Discriminator	1	$(7, 100, 100)$		$(64, 35, 35)$				1
	2∗	$(7, 100, 100)$		$(64, 44, 44)$	$(128, 16, 16)$			1
	3	$(7, 100, 100)$		$(64, 48, 48)$	$(128, 122, 22)$	$(256, 9, 9)$		1
	4	$(7, 100, 100)$		$(64, 49, 49)$	$(128, 24, 24)$	$(256, 11, 11)$	$(512, 5, 5)$	1
Generator	1∗	106	106	$(64, 35, 35)$				$(1, 100, 100)$
	2	106	106	$(128, 16, 16)$	$(64, 44, 44)$			$(1, 100, 100)$
	3	106	106	$(256, 9, 9)$	$(128, 22, 22)$	$(64, 48, 48)$		$(1, 100, 100)$
	4	106	106	$(513, 6, 6)$	$(256, 13, 13)$	$(128, 26, 26)$	$(64, 51, 51)$	$(1, 100, 100)$

Table S3: Candidate models for functional output regression in the prediction of optical absorption spectra. Asterisks denote the best-performing model selected through model validation.

Module	Dataset	Input	Layer1	Layer2	Layer3	Layer4	Output
1	Dataset I	1024	512				128
	Dataset II	1024	512				128
	USGS	1024	640				256
2	Dataset I	1024	512	256			128
	Dataset II	1024	512	256			128
	USGS	1024	768	512			256
3	Dataset I	1024	896	512	256		128
	Dataset II*	1024	896	512	256		128
	USGS*	1024	832	640	448		256
4	Dataset I*	1024	896	640	512	256	128
	Dataset II	1024	896	640	512	256	128
	USGS	1024	873	718	564	410	256

Table S4: Candidate models for functional output regression in the microstructure prediction. Asterisks denote the best-performing model selected through model validation.

# of layers	Input	Layer0 (Linear)	sampling + noise	Layer1 (Conv)	Layer2 (Conv)	Layer3 (Conv)	Layer4 (Conv)	Output
1	6	2	7	$(500, 16, 16)$				$(1, 32, 32)$
2	6	2	7	$(700, 8, 8)$	$(350, 16, 16)$			$(1, 32, 32)$
3	6	2	7	$(750, 4, 4)$	$(500, 8, 8)$	$(250, 16, 16)$		$(1, 32, 32)$
4*	6	2	7	$(800, 2, 2)$	$(600, 4, 4)$	$(400, 8, 8)$	$(200, 16, 16)$	$(1, 32, 32)$

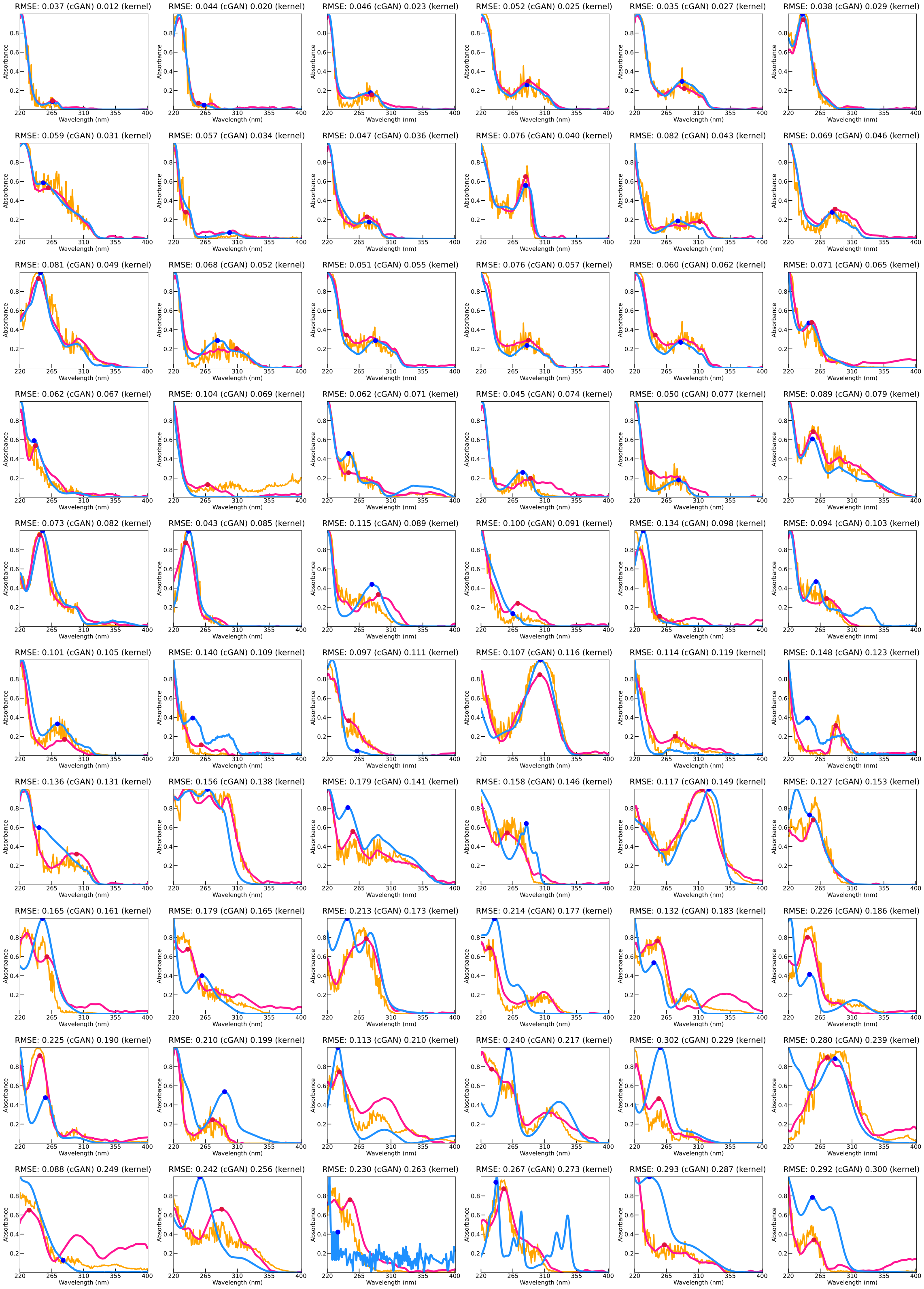

Figure S3: Dataset I: Predicted absorption spectra of the trained cGAN (orange) and functional output kernel regression (pink) for 60 randomly selected test molecules in Dataset I with their experimental spectral profiles (blue).

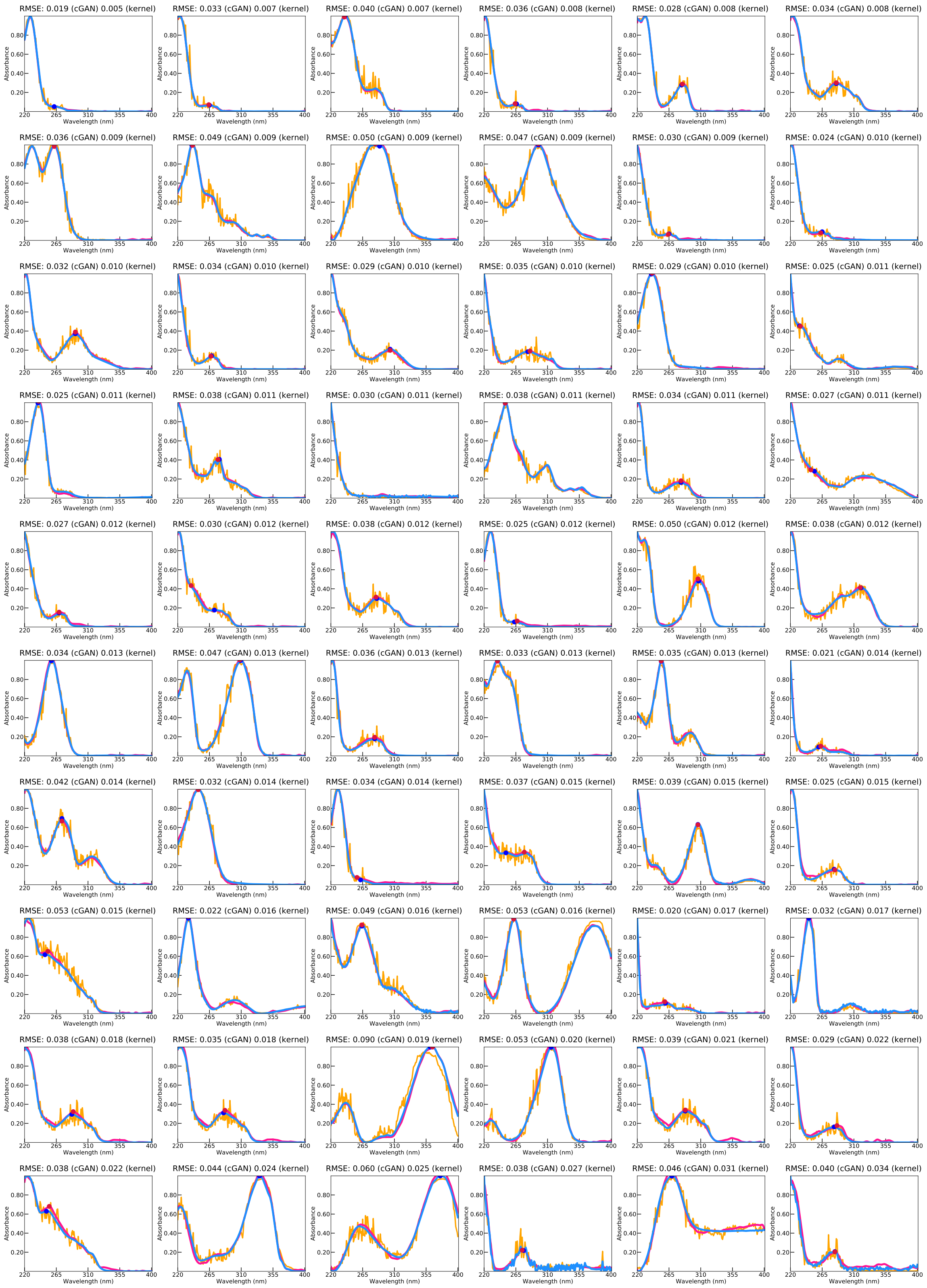

Figure S4: Dataset I: Results of fitting to training data for cGAN (orange) and functional output kernel regression (pink) with their experimental spectral profiles (blue).

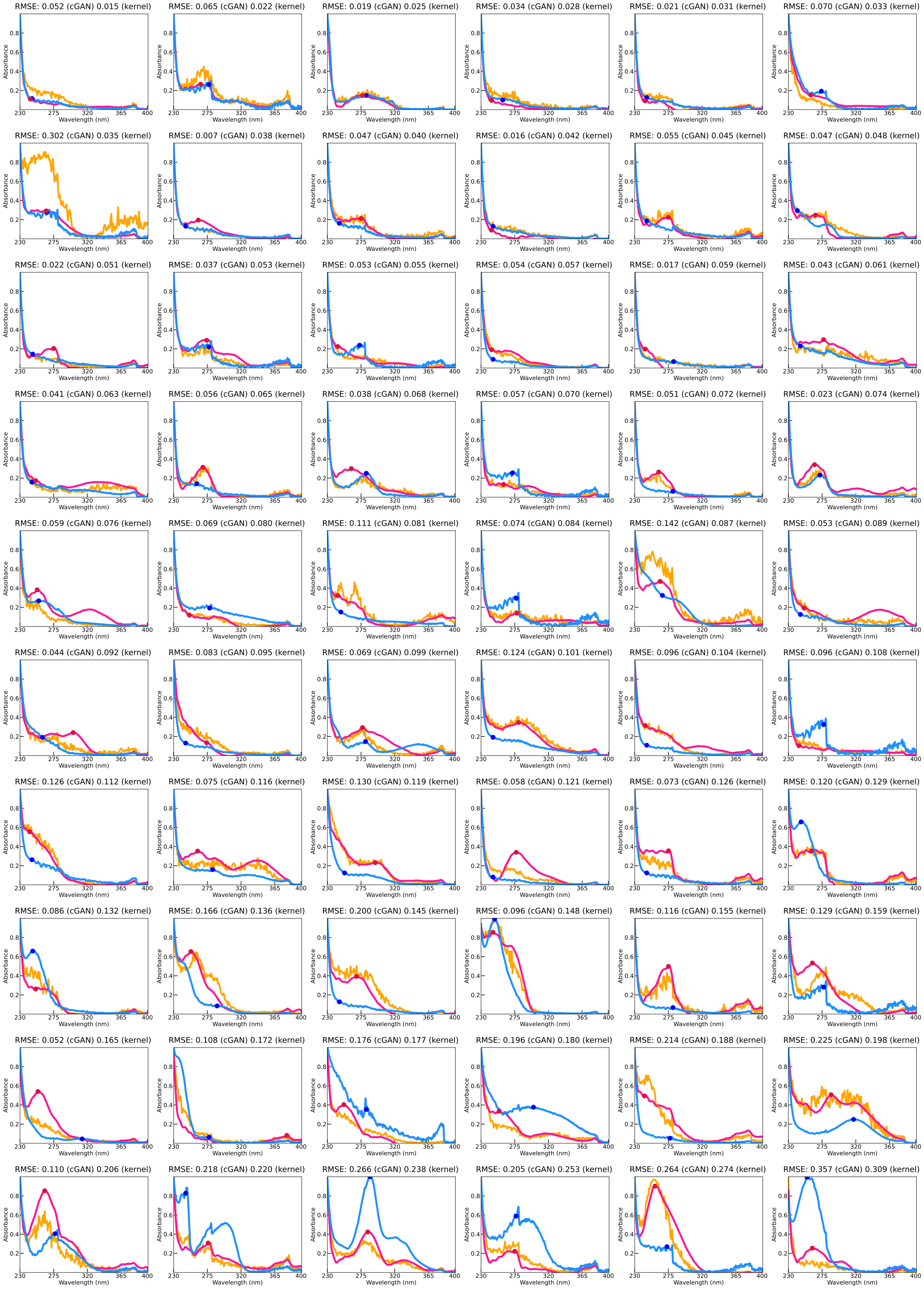

Figure S5: Dataset II: Predicted absorption spectra of the trained cGAN (orange) and functional output kernel regression (pink) for 60 randomly selected test molecules in Dataset II with their experimental spectral profiles (blue).

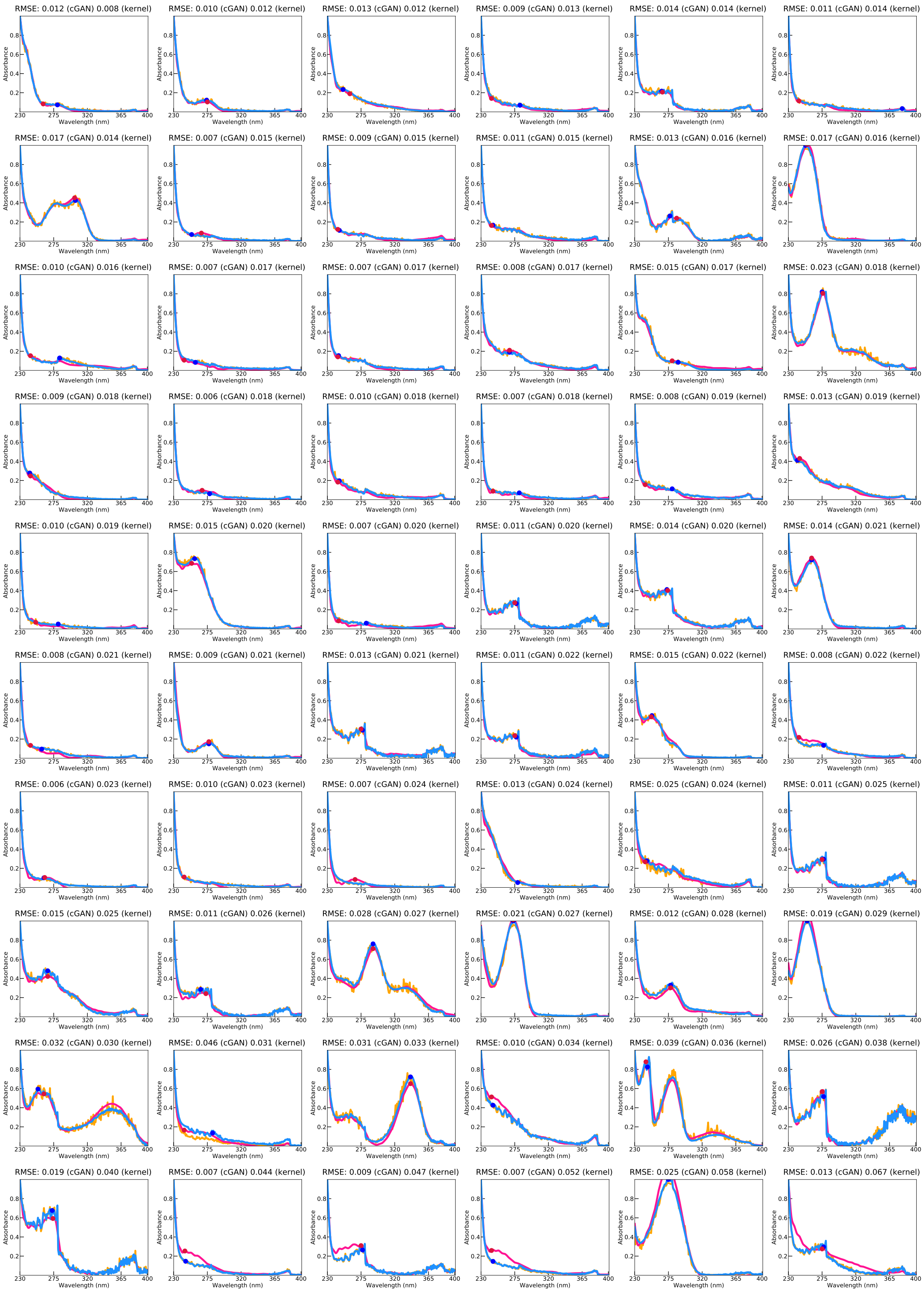

Figure S6: Dataset II: Results of fitting to training data for cGAN (orange) and functional output kernel regression (pink) with their experimental spectral profiles (blue).

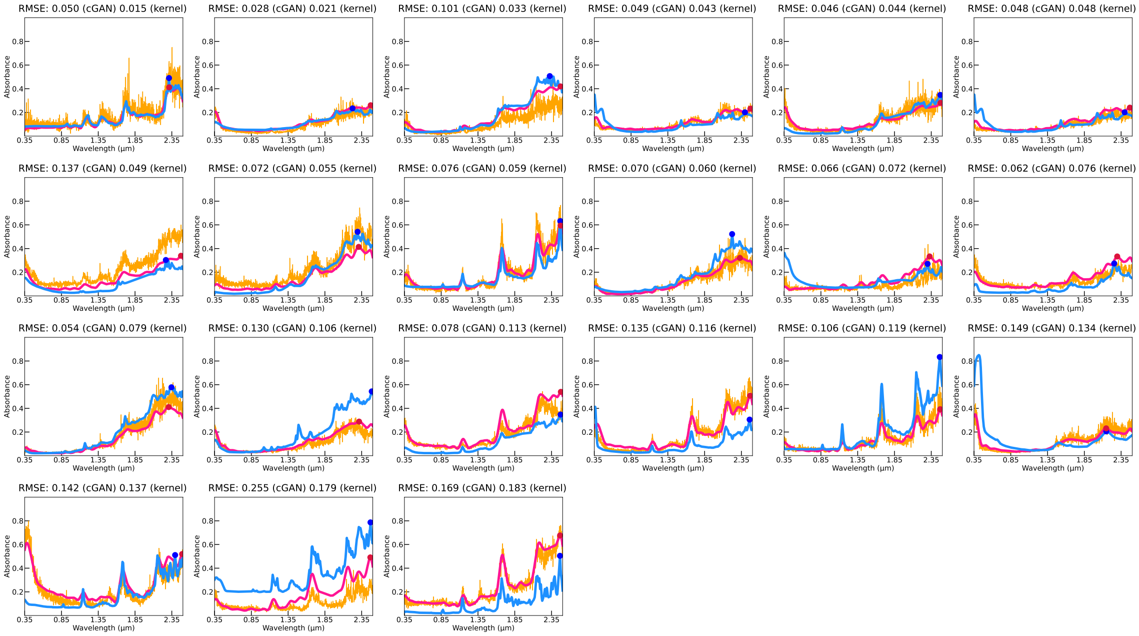

Figure S7: USGS spectral library: Predicted absorption spectra of the trained cGAN (orange) and functional output kernel regression (pink) for 21 test molecules in the library with their experimental spectral profiles (blue).

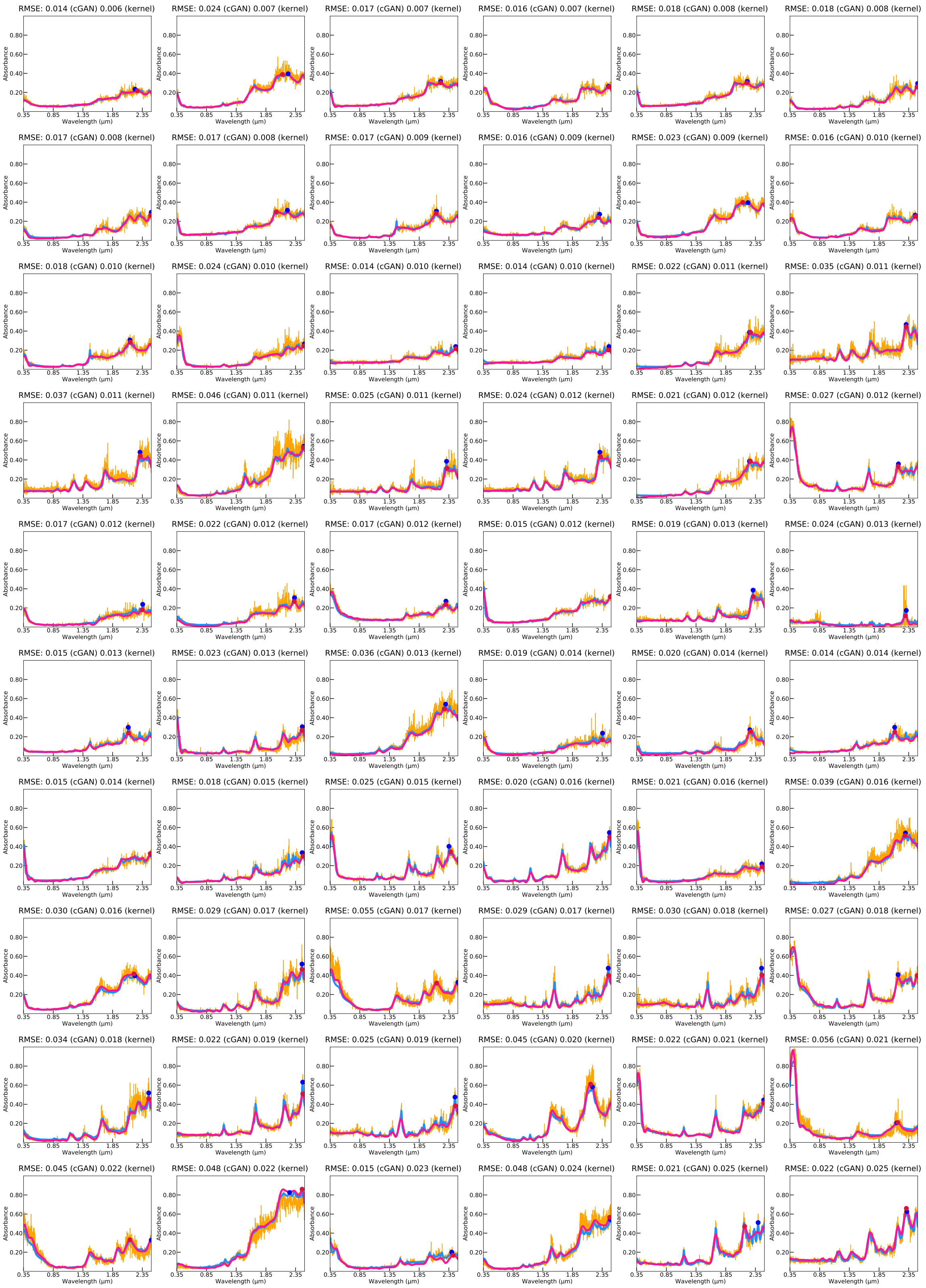

Figure S8: USGS spectral library: Results of fitting to training data for cGAN (orange) and functional output kernel regression (pink) with their experimental spectral profiles (blue).

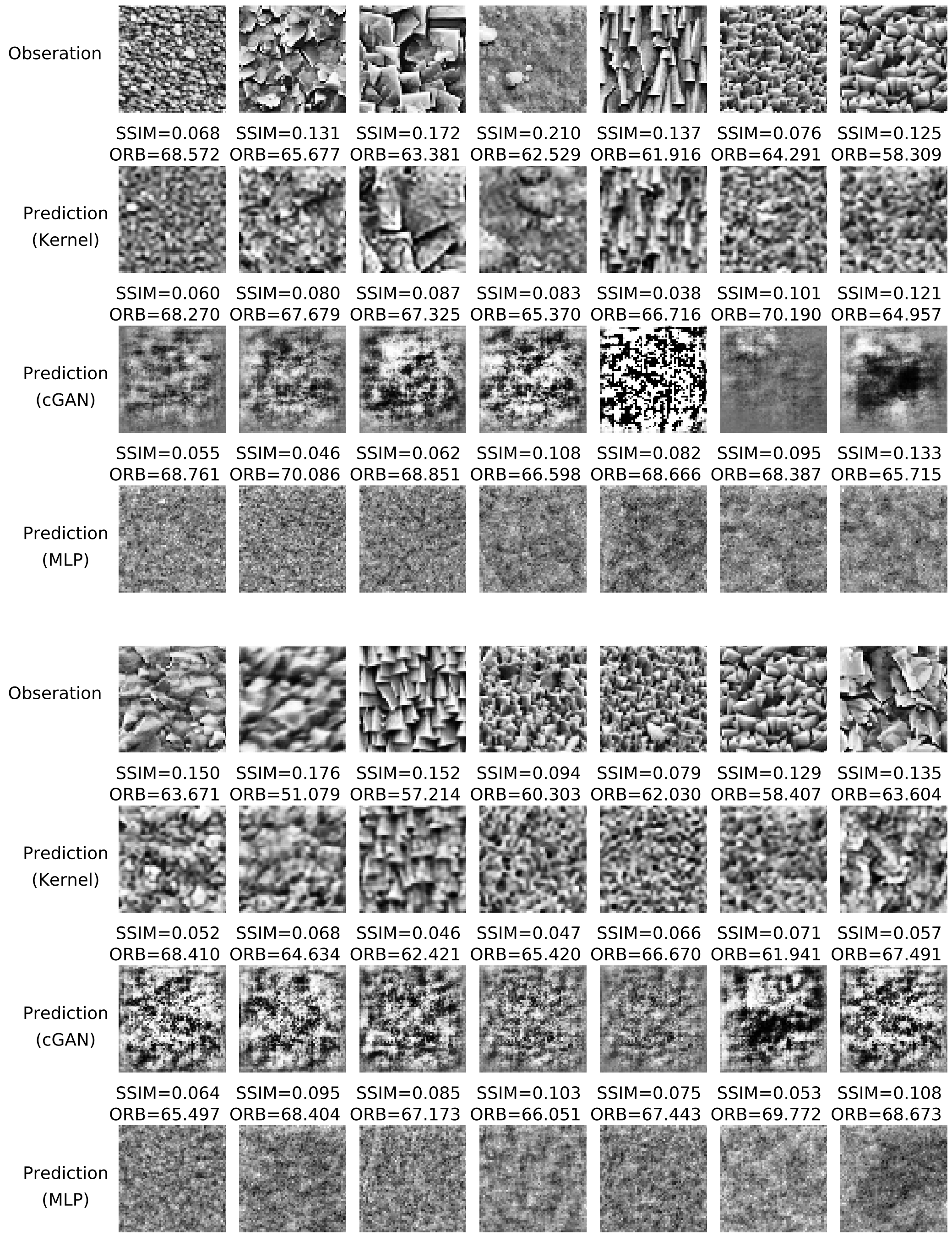

Figure S9: Microstructure prediction: Predicted images of the trained cGAN, functional output kernel regression and MLPs, are shown for 14 test instances with their experimental SEM images.

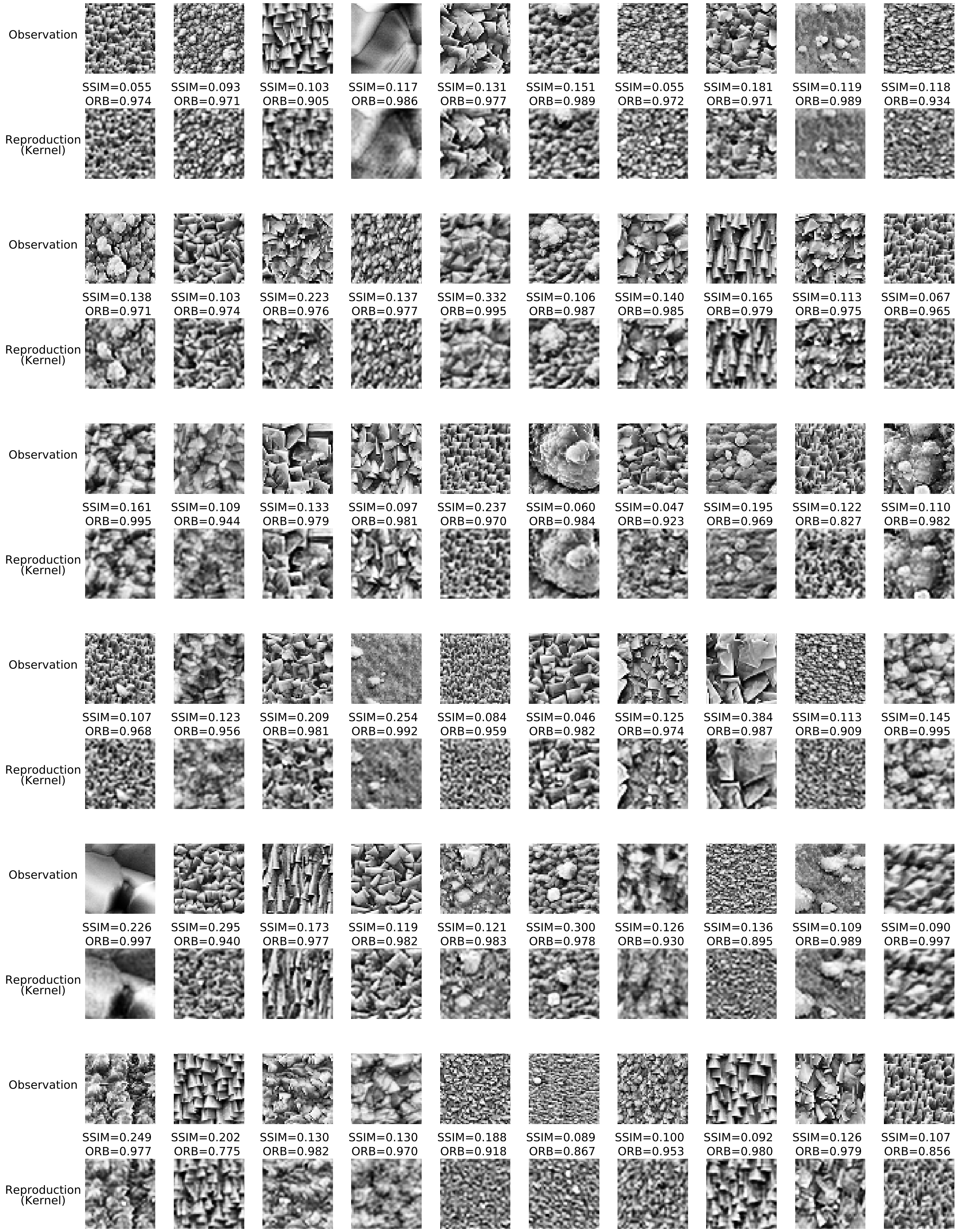

Figure S10: Reproduction of training data: Reproduced images of trained functional output kernel regression are shown for 60 cases with their experimental SEM images.
